# The PRC2-binding long non-coding RNAs in human and mouse genomes are associated with predictive sequence features

**DOI:** 10.1038/srep41669

**Published:** 2017-01-31

**Authors:** Shiqi Tu, Guo-Cheng Yuan, Zhen Shao

**Affiliations:** 1 Chinese Academy of Sciences Key Laboratory of Computational Biology, Collaborative Innovation Center for Genetics and Developmental Biology, Chinese Academy of Sciences-Max Planck Society Partner Institute for Computational Biology, Shanghai Institutes for Biological Sciences, Chinese Academy of Sciences, Shanghai 200031, China; 2 Graduate University of Chinese Academy of Sciences, Beijing 100049, China; 3 Department of Biostatistics and Computational Biology, Dana-Farber Cancer Institute, Boston, MA 02215, USA; 4 Department of Biostatistics, Harvard T.H. Chan School of Public Health, Boston, MA 02115, USA; 5 Harvard Stem Cell Institute, Cambridge, MA 02138, USA

## Abstract

Recently, long non-coding RNAs (lncRNAs) have emerged as an important class of molecules involved in many cellular processes. One of their primary functions is to shape epigenetic landscape through interactions with chromatin modifying proteins. However, mechanisms contributing to the specificity of such interactions remain poorly understood. Here we took the human and mouse lncRNAs that were experimentally determined to have physical interactions with Polycomb repressive complex 2 (PRC2), and systematically investigated the sequence features of these lncRNAs by developing a new computational pipeline for sequences composition analysis, in which each sequence is considered as a series of transitions between adjacent nucleotides. Through that, PRC2-binding lncRNAs were found to be associated with a set of distinctive and evolutionarily conserved sequence features, which can be utilized to distinguish them from the others with considerable accuracy. We further identified fragments of PRC2-binding lncRNAs that are enriched with these sequence features, and found they show strong PRC2-binding signals and are more highly conserved across species than the other parts, implying their functional importance.

Polycomb group (PcG) proteins are important epigenetic regulators in development and disease^1^,^2^. In mammalian cells, although quite a few transcription factors has been found to be linked with the chromatin binding and function of PcG proteins^1^,^3^,^4^,^5^,^6^, yet the underlying mechanisms controlling their site-specific chromatin recruitment remain incompletely understood. Since the identification of XIST and HOTAIR^7^,^8^, non-coding RNA-mediated recruitment of Polycomb repressive complex 2 (PRC2) has become a plausible, potentially sequence-dependent mechanism for Polycomb protein and H3K27me3 target regulation^1^. Recently, a set of RNA coimmunoprecipitation and chip hybridization (RIP-chip) experiments were published, which examined the expression and function of hundreds of lncRNAs in three different human cell types, and found more than 200 of them can physically interact with the core subunits of PRC2^9^. This result provided the first population-scale evidence of the interaction between lncRNA and PRC2.

Although a number of models have been proposed to elucidate how lncRNAs interact with their protein partners, especially chromatin remodeling factors, and participate in epigenetic regulations^10^,^11^,^12^, only a few large-scale RIP experiments have been published^9^,^13^, which makes it extremely difficult to study the role of interactions between lncRNAs and chromatin remodeling factors across different cell types. In particular, the precise mechanism through which lncRNAs may be targeted by chromatin remodeling factors, such as Polycomb proteins, is unclear. For example, it remains under debate whether PRC2 binds to RNA in a sequence dependent manner^14^,^15^,^16^,^17^, and it has been proposed that promiscuous and specific RNA binding may both exist for PRC2^15^. Moreover, quite a number of PRC2-binding lncRNAs have been discovered in human and mouse genomes^7^,^8^,^9^,^13^, but it is still not clear whether the mechanisms mediating *in vivo* PRC2-lncRNA interactions are evolutionarily conserved^15^.

In order to address these important questions, we carry out a systematic analysis of the DNA sequence patterns associated with PRC2-binding lncRNAs in both human and mouse genomes. In particular, we have developed a new computational pipeline for analyzing the composition of long DNA and RNA sequences of variable length using a Markov-chain based approach^18^. It considers each sequence as a series of transitions between adjacent nucleotides and uses the frequency of observing each possible transition to characterize the composition of this sequence. Through application of this pipeline to the PRC2-binding and non-binding lncRNAs identified from publicly available RIP data in human and mouse, we discovered a number of transitions that are differentially favored by these two classes of lncRNAs as the sequence features associated with PRC2-lncRNA interactions. By mapping all possible transitions to a complete quad-tree, we found a considerable fraction of transitions favored by PRC2-binding lncRNAs are located in consecutive paths, and these transitions are more likely to be simultaneously favored by human and mouse PRC2-binding lncRNAs than the others. We further built prediction models using the sequence features of PRC2-binding lncRNAs as predictors, which could distinguish these lncRNAs from the others with considerable accuracy. Remarkably, the fragments of PRC2-binding lncRNAs that are highly enriched with these sequence features show significant conservation across species, indicating the importance of these fragments.

## Results

### PRC2-lncRNA interactions in human are associated with significant sequence specificity

**Figure 1:**
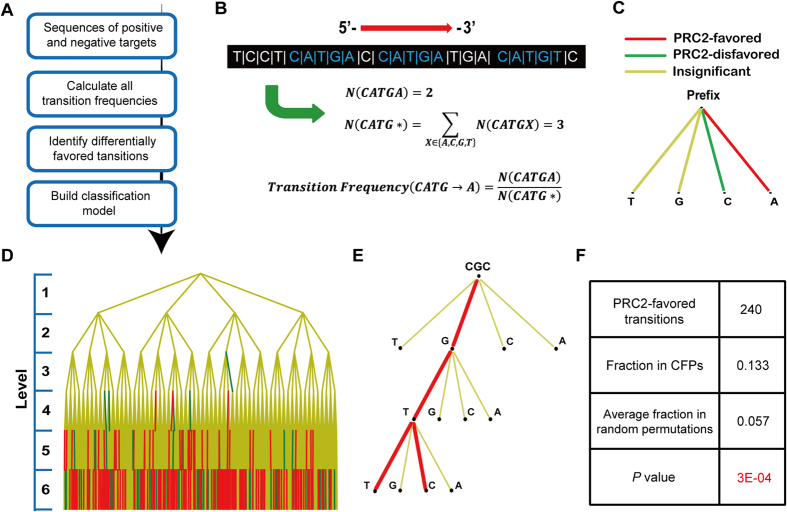
Analysis of the sequence features of human PRC2-binding lncRNAs. (**A**) Workflow of the sequence composition analysis pipeline. (**B**) Calculation of transition frequency, which is defined as the frequency of observing a transition in the given sequence (here order-4 transition CATG→A is used as an example). (**C**) A building block of quad-tree comprised of 4 transitions with the same prefix. Each line represents a transition and the color indicates whether the transition is significantly favored or disfavored by human PRC2-positive lncRNAs. (**D**) The complete quad-tree of height 6 constituted by all possible transitions of order 0–5 (placed on level 1-6 accordingly). Particularly, the root is an empty string as the prefix of 4 order-0 transitions. (**E**) A branch cut from the quad-tree shown in (**D**), which starts from level 3 and contains two consecutively favored paths (CFPs) CGC→G→T→T and CGC→G→T→C. (**F**) Summary statistics of the CFPs observed in (**D**), which suggest the human PRC2-favored transitions significantly prefer to connect with each other and form CFPs.

Figure 1A shows an overview of our computational pipeline for sequence composition analysis. It takes two distinct groups of sequences as input, e.g. the DNA sequences of genes that are associated and not associated with a specific biological function. In this pipeline, a systematic analysis is applied to study the compositional patterns of input sequences by modeling each sequence as a Markov chain^18^,^19^,^20^, which can be dissected into a series of transitions between adjacent nucleotides (Fig. 1B). To avoid arbitrarily selecting the exact order of Markov chain model, all possible transitions of order 0 through m are utilized (here we chose m = 5, which led to 5460 possible transitions in total). Next, transitions differentially favored by two sequence groups are selected as their sequence features (Fig. 1C; see Methods). Finally, a classification model is constructed by applying Bayesian additive regression trees (BART)^21^ analysis to test whether these sequence features can be used to predict the group label of each sequence.

To investigate the role of lncRNAs’ sequence composition in mediating their interactions with PRC2, we first collected 261 human lncRNAs that can physically interact with the core subunits of PRC2 in three human cell types from Khalil *et al*.^9^, together with 227 lncRNAs that were expressed in these cell types but failed to show detectable interaction with PRC2. These two groups of lncRNAs were labeled as human PRC2-positive and PRC2-negative lncRNAs, respectively. Next, we applied our pipeline to compare their sequence composition, with the purpose to uncover the underlying sequence features associated with PRC2-lncRNA interactions. As a result, we identified 240 transitions that are significantly favored by human PRC2-positive lncRNAs compared to PRC2-negative ones, together with 87 transitions significantly disfavored by them (using *P*-value < 0.05 as cutoff), and named them as human PRC2-favored and disfavored transitions, respectively. To make a global visualization of these transitions, we constructed a complete quad-tree of height 6 comprised of all 5460 possible transitions of order 0 through 5, which were placed on level 1 through 6 of the tree accordingly. Here the PRC2-favored and disfavored transitions were specially colored as red and green, respectively (Fig. 1C,D).

Besides serving as a platform for visualization, the quad-tree can also be utilized to test whether the selected transitions form a nontrivial subset of all 5460 possible transitions, by inspecting their distribution on the tree. We first examined the number of PRC2-favored and disfavored transitions on each level, and found the vast majority of these transitions are on level 6 (Table 1 and Supplementary Table S1). To estimate the significance of this observation, we generated 1000 sets of randomized PRC2-positive and PRC2-negative lncRNAs, each of which was derived by randomly shuffling the original group labels of 488 human PRC2-positive and PRC2-negative lncRNAs (see Methods), and repeated the same feature selection process on each randomized lncRNA set. By this means, only the number of PRC2-favored transitions on level 6 was found to be significantly higher than that observed from randomized lncRNA sets (*P* = 0.007, Table 1). Furthermore, although the selected transitions showed a rather sparse distribution on quad-tree, we still observed that these transitions, especially the PRC2-favored ones, tend to connect with each other across adjacent levels and form consecutive paths. To validate this finding, we define consecutively favored/disfavored paths (CFPs/CDPs) as the consecutive paths on quad-tree that are completely constituted by PRC2-favored/disfavored transitions (Fig. 1E and Supplementary Fig. S1A), respectively. Interestingly, a considerable fraction of PRC2-favored transitions are located in CFPs (13.3%, Fig. 1F), and this value is significantly higher than that observed in random simulations, in which all the PRC2-favored transitions were randomly re-distributed on each level (*P* = 3E-4 by permutation test, see Methods). On the other hand, PRC2-disfavored transitions only exhibited a weak enrichment in CDPs compared to that expected by chance (9.2% and *P* = 0.023, Supplementary Fig. S1B).

To understand why PRC2-favored transitions prefer to form consecutive paths, we additionally applied our pipeline to analyze the sequence features associated with transcription factor CTCF’s DNA binding in human cells (see Supplementary text for details), as the sequence specificity of this interaction is largely known^22^. Strikingly, the vast majority of CTCF-favored transitions identified by our pipeline are located in CFPs and, particularly, 52 CFPs are of full length. We compared a representative full-length CFP with CTCF’s binding motif obtained from JASPAR database^23^, and found the 6-mer formed by this full-length CFP can be well matched with the motif (Supplementary Fig. S1C). Inspired by this observation, we calculated the motif score of each full-length path, which is defined to measure the similarity between the 6-mer formed by all the 6 transitions on this path and CTCF’s binding motif (see Supplementary text for details). Interestingly, strong correlation was observed between the length of the longest CFP lying on each full-length path and the motif score of this path (Supplementary Fig. S1D), suggesting the preference of those favored transitions to form consecutive paths may be intrinsically connected with the sequence specificity mediating the binding of corresponding proteins and the CFPs observed in this study can be of biological importance.

### The sequence features of human PRC2-binding lncRNAs are predictive of PRC2-lncRNA interactions

**Figure 2:**
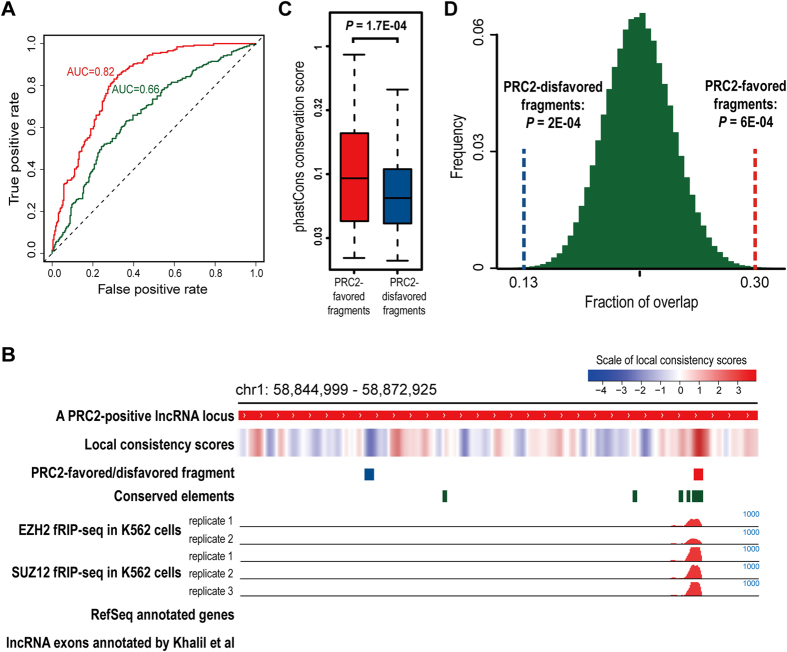
Prediction of the PRC2-lncRNA interactions in human genome based on transition frequencies. (**A**) ROC curves and corresponding AUC values of the prediction models built by the non-blind CV (red line) and the fully blind method (green line) in predicting human PRC2-binding lncRNAs. (**B**) A representative PRC2-positive lncRNA locus. Here its PRC2-favored and disfavored fragment are indicated by the red and blue bar, respectively, and the red tracks in the middle show the fRIP-seq read counts of EZH2 and SUZ12 in human K562 cell line. (**C**) Boxplot of the average PhastCons conservation scores of the PRC2-favored and disfavored fragments identified from human PRC2-binding lncRNAs. (**D**) Distribution of the fraction of the 500 bp fragments randomly selected from human PRC2-binding lncRNAs that overlap with the conserved elements. Here the distribution was draw from 10^5^ times of random sampling and dash lines represent the fraction of PRC2-favored/disfavored fragments that overlap with the conserved elements.

In order to evaluate whether the sequence features identified by our pipeline can be used to predict PRC2-binding lncRNAs, we took the frequencies of all human PRC2-favored and disfavored transitions as predictors, and employed BART analysis^21^ to fit a prediction model. Based on a standard 10-fold cross-validation (CV) process, we found this model is able to distinguish human PRC2-positive lncRNAs from the negative ones with good accuracy, and the area under the receiver operating characteristic (ROC) curve (AUC) is 0.82 (Fig. 2A), which is close to that achieved by the prediction model published previously^24^. In addition, we also adopted a more stringent approach of model building, which is called as fully blind method here. Similar to the original 10-fold CV process, all human PRC2-positive and PRC2-negative lncRNAs are divided into 10 subgroups and at each cross-validation step, only one subgroup is selected as the testing set, leaving the other 9 subgroups as training set. The key difference of the fully blind method is that predictor selection is repeatedly performed at each cross-validation step and only lncRNAs in the training set can be used to identify PRC2-favored and disfavored transitions as predictors (see Methods), which means the predictors used at each step may not be exactly the same as the 240 PRC2-favored and 87 PRC2-disfavored transitions that were used as predictors in the original 10-fold CV process (it will be called as “non-blind CV” from now on in this study). In this way, human PRC2-binding lncRNAs were predicted with moderate accuracy (AUC = 0.66). More specifically, 76% of the top 261 lncRNAs predicted by the non-blind CV approach are true PRC2-positive ones, and this fraction decreased to 66% for the top 261 lncRNAs predicted by the fully blind approach. To explain why prediction models built by these two methods exhibited distinct performance, we drew the ROC curve for each of the 10 lncRNA subgroups separately and calculated the corresponding AUC value. Remarkably, compared to the non-blind CV method, the AUC values of 10 lncRNA subgroups got from the prediction model built by fully blind method exhibited much higher variation, with a range from 0.53 to 0.8 (Supplementary Fig. S2A). On the other hand, we devised two empirical classification models using the non-blind CV and also the fully blind method, respectively, but without involving BART to perform sophisticated model training (see Supplementary text for details). By comparing the performance of these empirical models on the same set of human lncRNAs, it could be clearly viewed that whether or not to exclude lncRNAs in the testing set from predictor selection can strongly affect prediction accuracy (Supplementary Fig. S2B,C). Since the fully blind method is more stringent, we think it’s better to use prediction models built by this method to infer how accurately PRC2-binding lncRNAs can be predicted by their sequence composition.

Next, we further investigated the distribution of the sequence features of human PRC2-binding lncRNAs along their gene bodies. Following this direction, each PRC2-positive lncRNA was scanned by a sliding window of size 500 bp and a local consistency score was assigned to the sequence fragment in the window, which is defined as the sum of the frequencies of all PRC2-favored transitions in this sequence fragment minus those of all PRC2-disfavored ones. In this way, sequences with high consistency scores should be highly enriched for PRC2-favored transitions and also depleted of PRC2-disfavored ones. Interestingly, these lncRNAs exhibit highly non-uniform local consistency scores along their gene bodies, and some fragments of them have clearly higher scores than the others (Fig. 2B). Inspired by this finding, we defined the fragment with the highest/lowest consistency score in each human PRC2-positive lncRNA as its PRC2-favored/disfavored fragment (Fig. 2B), respectively. To know whether they can be potentially important for PRC2-lncRNA interactions, we examined the RNA binding of PRC2 on these fragments as well as their conservation level across vertebrate genomes (Fig. 2B). For the first analysis, we incorporated a recently published RIP-seq dataset of PRC2 core subunit EZH2 and SUZ12 in K562 human leukemia cell line^14^, and calculated the RIP-seq read density at each PRC2-favored and disfavored fragment. Interestingly, binding of EZH2 and SUZ12 at PRC2-favored fragments was found to be stronger than that at PRC2-disfavored ones (Supplementary Fig. S2D). Meanwhile, we also observed PRC2-favored fragments have significantly higher average conservation scores than PRC2-disfavored ones (*P* = 1.7E-04 by paired t-test; Fig. 2C). More explicitly, 30% of PRC2-favored fragments overlap with conserved elements^25^,^26^, which is significantly higher than that of the 500 bp fragments randomly selected from the same lncRNAs (*P* = 6E-04), and this fraction for PRC2-disfavored fragments is only 13% (*P* = 2E-04; Fig. 2D).

To check whether the high conservation level of PRC2-favored fragments are directly linked with the aggregation of sequence features associated with PRC2-lncRNA interactions, we again took the 1000 sets of randomized PRC2-positive and PRC2-negative lncRNAs, and reselected a group of pseudo PRC2-favored and disfavored fragments for each set of randomized PRC2-positive lncRNAs using the pseudo PRC2-favored and disfavored transitions associated with this randomized lncRNA set, which were identified by comparing the sequence composition of these lncRNAs with the corresponding randomized PRC2-negative lncRNAs. Then, the same analyses as shown in Fig. 2C,D were applied to each set of pseudo PRC2-favored and disfavored fragments identified from the randomized PRC2-positive lncRNAs. Remarkably, when comparing the average conservation scores of pseudo PRC2-favored and disfavored fragments, only 1.7% of the 1000 randomized lncRNA sets achieved *P*-values lower than that shown in Fig. 2C (Supplementary Fig. S2E,F), which is taken as an empirical estimate of the false positive rate (FPR) of the test conducted in Fig. 2C (Supplementary Fig. S2F). Similarly, we calculated the *P*-value for the overlap between each set of pseudo PRC2-favored fragments and conserved elements by comparing with the fragments randomly selected from the same lncRNAs, and found only 0.8% of the 1000 sets of pseudo PRC2-favored fragments got *P*-values lower than that shown in Fig. 2D (Supplementary Fig. S2F). Taken together, these findings indicate the PRC2-favored fragments, which are highly enriched with sequence features associated with PRC2-lncRNA interactions, are generally more conserved than the other parts of the lncRNAs they belong to, and, thus, are more likely to be of functional importance.

### Comparison of the sequence features of human and mouse PRC2-binding lncRNAs

**Figure 3:**
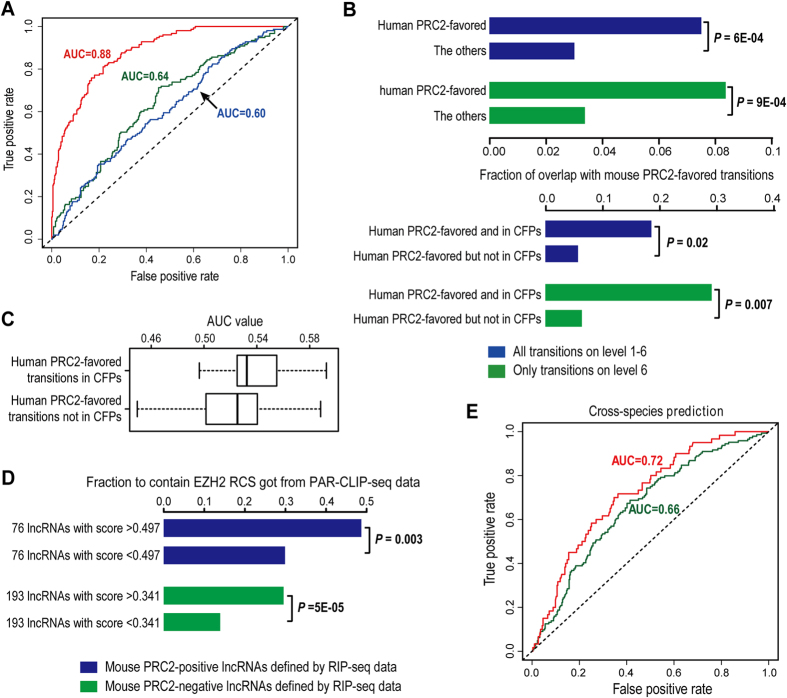
Human PRC2-favored transitions in CFPs are more likely to be also favored by mouse PRC2-binding lncRNAs than the others. (**A**) ROC curves and corresponding AUC values of different prediction models in predicting mouse PRC2-binding lncRNAs. The red and green curve correspond to mouse prediction models built by the non-blind CV and the fully blind method, respectively, in which mouse PRC2-positive and PRC2-negative lncRNAs were used for predictor selection and model training. The blue curve corresponds to the human prediction model using human PRC2-positive and PRC2-negative lncRNAs for predictor selection and model training. (**B**) Fractions of different groups of transitions that are identified as mouse PRC2-favored transitions. Here, the *P*-values were computed by right-tailed Fisher’s exact test based on hypergeometric distribution. (**C**) Boxplot of the AUC values of human PRC2-favored transitions in predicting mouse PRC2-binding lncRNAs. Here the human PRC2-favored transitions are divided into 2 groups based on whether or not they are located in CFPs, and the AUC value of a transition is calculated by directly using its frequency in each sequence as the prediction score of this sequence. (**D**) Fraction of mouse PRC2-positive and PRC2-negative lncRNAs that contain EZH2 RCS identified from PAR-CLIP-seq data. Here each group of lncRNAs were split into two subgroups of equal size by the median of their cross-species prediction scores derived from the prediction model trained with human lncRNAs, and the *P*-values were calculated by right-tailed Fisher’s exact test to measure whether the subgroup of lncRNAs with high prediction scores are significantly more likely to contain EZH2 RCS compared to the subgroup with low prediction scores. (**E**) ROC curve and corresponding AUC value of the human prediction model in predicting mouse RCS-containing lncRNAs from the RCS-null ones (green), and also that in predicting high-confidence mouse PRC2-positive lncRNAs from high-confidence mouse PRC2-negative ones (blue).

The core subunits of PRC2 as well as their roles in transcriptional repression are highly conserved from Drosophila to mammals^1^. Besides, interactions between PRC2 and lncRNAs are detected in both human and mouse cells, and some of them are shared between two species^7^,^8^. Thus, it would be interesting to know whether the PRC2-binding lncRNAs in human and mouse genomes tend to share common sequence features, despite that the sequences of lncRNAs are known to be generally much less conserved than protein-coding genes^27^. To answer this question, we first studied a RIP-seq dataset of EZH2 generated from mouse embryonic stem cells (mESCs)^13^. Based on this dataset and the mouse lncRNAs that were discovered in parallel with the human ones used in this study^28^, we obtained 153 mouse lncRNAs having physical interactions with EZH2 in mESCs, together with 387 lncRNAs that are expressed in mESCs but failed to show detectable interaction with EZH2, which were labeled as mouse PRC2-positive and PRC2-negative lncRNAs, respectively (see Supplementary text for details). Subsequently, the same sequence composition analysis was applied to these lncRNAs, and 175 mouse PRC2-favored transitions as well as 116 PRC2-disfavored ones were identified as the sequence features of mouse PRC2-binding lncRNAs. Then, we used the frequencies of these transitions as predictors and employed BART analysis to fit a prediction model of mouse PRC2-binding lncRNAs. Similar to what we observed from human lncRNAs, prediction model built by the fully blind method exhibited moderate accuracy (AUC = 0.64), clearly lower than the model built by the non-blind CV method (AUC = 0.88, Fig. 3A). Meanwhile, we fitted a prediction model using all human PRC2-positive and PRC2-negative lncRNAs as training set and all the human PRC2-favored and disfavored transitions as predictors, and applied this model on mouse lncRNAs to perform cross-species prediction. Interestingly, by this means mouse PRC2-positive lncRNAs can be distinguished from the PRC2-negative ones with considerable accuracy (AUC = 0.60, Fig. 3A). Since in the cross-species prediction only human lncRNAs were involved in predictor selection, its performance should be compared with the prediction model trained with mouse lncRNAs using the fully blind method. Thus, we speculate some sequence features are shared between human and mouse PRC2-binding lncRNAs.

Inspired by this hypothesis, we analyzed the overlap between the sequence features of human and mouse PRC2-binding lncRNAs. In general, only 10% of the PRC2-favored transitions are shared between human and mouse (18 of 240/175, Supplementary Fig. S3A), though still significantly higher than expected by chance (Fig. 3A). As we have shown, human PRC2-favored transitions prefer to be located on level 6 of the quad-tree and connect with each other to form CFPs. It’s reasonable to pay special attentions to these transitions. Interestingly, being on level 6 itself doesn’t significantly increase the likelihood of human PRC2-favored transitions being also favored by mouse PRC2-binding lncRNAs. However, 19% of the 32 human PRC2-favored transitions located in CFPs remained to be mouse PRC2-favored ones (which means 1/3 of the PRC2-favored transitions shared between human and mouse are located in human CFPs), and this fraction increases to 29% for the human PRC2-favored transitions located on level 6 and also in CFPs (Fig. 3B). Moreover, we tried building cross-species prediction models using all human PRC2-favored transitions and only those located in CFPs as predictors, respectively, and tested the performance of these models on mouse lncRNAs. Of note, they achieved AUC values (0.61 and 0.59) very close to the cross-species prediction model using all human PRC2-favored and disfavored transitions as predictors (Supplementary Fig. 3C). Then, we calculated the AUC value of each human PRC2-favored transition in predicting mouse PRC2-binding lncRNAs, which is computed by directly assigning the frequency of observing this transition in the sequence of each mouse lncRNA as the score of this lncRNA. Remarkably, the majority of human PRC2-favored transitions have AUC values greater than 0.5, which suggests these transitions tend to also be positively favored by mouse PRC2-binding lncRNAs, and, especially, the ones falling in CFPs achieved clearly higher AUC values than those out of CFPs (Fig. 3C). Thus, we conclude that the association between transitions falling in CFPs and PRC2-binding lncRNAs are more highly conserved between human and mouse than those out of CFPs. Again, these findings strongly support the biological significance of CFPs.

In recent years, *in vivo* UV light cross-linking and immunoprecipitation followed by high-throughput sequencing (CLIP-seq) experiments have also been widely used to study genome-wide protein-RNA interactions^29^,^30^,^31^,^32^. To make a more comprehensive assessment of our cross-species comparison of PRC2-binding lncRNAs, we additionally incorporated a recently published EZH2 PAR-CLIP-seq dataset in mESCs^31^. We obtained 13,764 putative RNA-contact sites (RCSs) of EZH2 from this dataset and mapped them to the mouse PRC2-positive and PRC2-negative lncRNAs we defined from the EZH2 RIP-seq data in mESCs. 39.2% of mouse PRC2-positive lncRNAs were found to contain at least one EZH2 RCS, and this fraction for mouse PRC2-negative ones is only 21.7% (Supplementary Fig. 3D, see Supplementary text for details), indicating a moderate consistency between these two datasets. Next, we divided both mouse PRC2-positive and PRC2-negative lncRNAs into two subgroups of equal size, based on their cross-species prediction scores derived from the prediction model trained with human lncRNAs. Interestingly, almost half of the mouse PRC2-positive lncRNAs with high cross-species prediction scores have EZH2 RCS identified from the PAR-CLIP-seq data (Fig. 3D), which is significantly higher than that of the PRC2-positive lncRNAs with low prediction scores (29.0%, *P* = 0.003 by Fisher’s exact test), indicating they are more likely to be true PRC2-binding lncRNAs. On the other hand, still a considerable faction of the mouse PRC2-negative lncRNAs with high cross-species prediction scores were found to contain EZH2 RCS (29.5%, Fig. 3D), which is also significantly greater than that of the PRC2-negative lncRNAs with low prediction scores (13.9%, *P* = 5E-5), implying many of them may actually have the potential to physically interact with PRC2 as predicted by their sequence similarity with the human PRC2-binding lncRNAs. Inspired by these findings, we defined high-confidence mouse PRC2-positive lncRNAs as the mouse PRC2-positive lncRNAs that also contain RCS of EZH2, and high-confidence mouse PRC2-negative lncRNAs as the mouse PRC2-negative lncRNAs with no EZH2 RCS. By taking only these high-confidence lncRNAs into account, we found the accuracy of cross-species prediction is even higher (AUC = 0.72, Fig. 3E), which strongly supports that a considerable proportion of the sequence patterns associated with PRC2-lncRNA interactions are shared between human and mouse.

### Compare the performance of prediction models based on transition and K-mer frequencies

**Figure 4:**
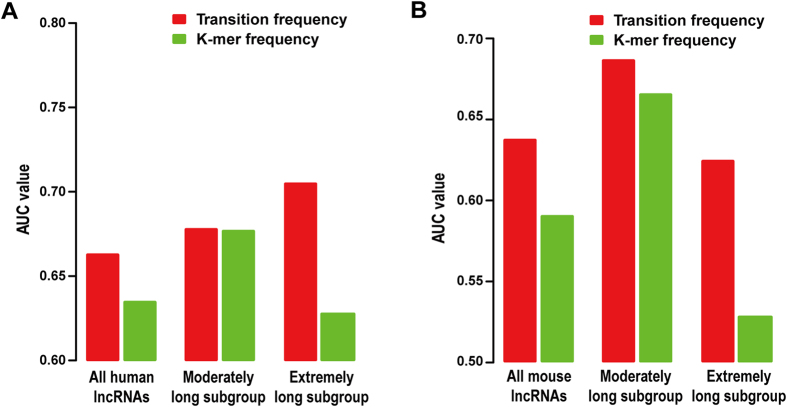
Compare the performance of prediction models based on K-mer and transition frequencies. (**A**,**B**) AUC values of the prediction models based on transition (red bars) or K-mer (blue bars) frequencies, which were trained and tested by the human (**A**) and mouse (**B**) lncRNAs, respectively. Here the prediction models were built by the fully blind method, and all human/mouse PRC2-positive and PRC2-negative lncRNA were separately divided into two subgroups of equal size according to their length, termed as the moderately long and the extremely long subgroup, to access the performance of these models on lncRNAs of different length.

In previous studies, the composition of a sequence was usually analyzed by counting the occurrence of different K-mers in it^18^,^33^, and typically the count of each K-mer would be further normalized by sequence length to represent the frequency of observing this K-mer in the sequence. Here, sequence composition analysis based on K-mer frequencies was also applied to study human PRC2-binding lncRNAs. Technically, we calculated the frequencies of all possible K-mers of length from 1 through 6 in the DNA sequence of each lncRNA, and searched for K-mers that occur in human PRC2-positive lncRNAs with significantly higher or lower frequencies than in the PRC2-negative ones (see Supplementary text for details). By using *P*-value < 0.05 as cutoff, 129/83 K-mers that are significantly over/under-represented in human PRC2-binding lncRNAs were identified, respectively. The prediction model using the frequencies of these K-mers as predictors showed slightly lower accuracy (AUC = 0.63 using the fully blind method) than the model based on transition frequencies (Fig. 4A). To assess the impact of lncRNA length, we separately divided the human PRC2-positive and PRC2-negative lncRNAs into two subgroups of equal size according to their length, which were named as moderately long and extremely long subgroup, respectively (the cutoff of lncRNA length to separate these two lncRNA subgroups is 18.6 kb for human PRC2-positive lncRNAs and 13.1 kb for PRC2-negative ones). Then, the performance of prediction was evaluated on the two subgroups separately. Interestingly, two prediction models achieved similar AUC values on the moderately long subgroup (Fig. 4A), while the model based on transition frequencies exhibited a better accuracy on the extremely long subgroup of lncRNAs (AUC = 0.70) than the one based on K-mer frequencies (AUC = 0.63; Fig. 4A). In addition, we also performed the same analysis on mouse lncRNAs and got a similar result (Fig. 4B), suggesting this finding is not specific to the human lncRNAs chosen by us and sequence composition analysis based on transition frequencies can have plausible performances on extremely long sequences.

It should be noted that besides finding the sequence features associated with PRC2-lncRNA interactions in mammalian cells, another main purpose of this study is to develop a new computational pipeline for sequence composition analysis based on splitting each sequence into transitions between adjacent nucleotides. We have demonstrated sequence composition analysis based on transition frequencies can have different downstream analyses from that based on K-mers, e.g. to examine the distribution of selected transitions on a quad-tree, which led to the identification of CFPs (Supplementary Fig. S1C and S4D). However, which of these two types of methods can have a superior performance highly depends on the context (a theoretical example is given in Supplementary text) as well as the implementation of these methods, since the analysis based on K-mers has been widely used for years and a large number of computation models have been developed to improve its performance and to extend its applications^18^.

## Discussion

In this study, we conducted a systematic sequence composition analysis on known PRC2-binding lncRNAs in both human and mouse genomes. To be noted, identifying characteristic sequence features from the lncRNAs associated with a specific biological function is important and also computationally challenging. One of the main reasons is that the length of lncRNA genes can be quite long and highly variable (here we chose to use the whole gene body of lncRNAs for sequence analysis, and a detailed explanation can be found in Supplementary text). In our case, the human lncRNAs used here are of size 33.4 ± 41.3 kb (average gene length ± standard deviation, the median length is 15.8 kb), and a large fraction of them may not be well annotated (two examples can be seen in Fig. 2B and Supplementary Fig. S2H), which makes the sequence analysis even more complicated. Here, we presented a new computational pipeline for analyzing the compositional patterns of long sequences, which considers each sequence as a series of transitions between adjacent nucleotides and can systematically search for transitions that occur in the sequences of interest with significantly different frequencies compared to the control sequences. Besides, the pipeline is incorporated with a set of computational analyses to visualize all candidate transitions using a complete quad-tree and then to dissect the distribution of selected transitions on the tree. Applying it to compare the sequences of PRC2-binding and non-binding lncRNAs in human and mouse genomes, we identified a large pool of transitions as features of PRC2-binding lncRNAs in each species, and found those transitions favored by PRC2-binding lncRNAs exhibit a significant preference to connect with each other and form CFPs on the quad tree, which seems not to be sufficiently appreciated by other similar studies. Interestingly, although the sequence features of PRC2-binding lncRNAs show a low overlap between human and mouse, the majority of human PRC2-favored transitions have AUC values higher than 0.5 in predicting mouse PRC2-binding lncRNAs, especially for those falling in CFPs (Fig. 3C). Although lncRNAs generally are thought to be poorly conserved^27^,^34^,^35^,^36^, our findings suggest PRC2-lncRNA interactions in mammalian cells are clearly associated with specific sequence patterns and these patterns tend to be conserved across species, which can be further supported by the good performance of cross-species predictions (Fig. 3D).

Another interesting aspect of our findings is that the sequence composition of lncRNAs can be highly complex along their gene bodies, which supports the hypothesis that such a great complexity might be necessary for its functions^37^. For example, as shown in Fig. 2D and Supplementary Fig. S2G,H, the sequence features of PRC2-binding lncRNAs showed a highly non-uniform distribution along their gene bodies and, particularly, some regions are significantly more enriched with these features than the other parts. Taking a step further, we recognized a set of fragments that are highly enriched with these features from human PRC2-binding lncRNAs, and found these fragments are significantly more highly conserved than the other parts of these lncRNAs, implying they may be potentially important for the function of these lncRNAs. This observation can provide a different viewpoint to understand the low conservation levels of lncRNAs in mammals, and implies evolutionary analyses can still serve as a useful tool for identifying functional elements of lncRNAs^38^. Taken together, our analysis indicates that, although the sequences of lncRNAs are of tremendous complexity, they still share quite a number of recurring patterns. Using these patterns as clues, our predictions based on global and local sequence compositions can serve as a useful guide for experimental biologists to investigate the potential connections between Polycomb group proteins and lncRNAs in a tissue-specific manner, and also to further dissect how these connections are established. For future studies, even more sophisticated models, e.g. nonhomogeneous Markov model^39^, may be employed to further understand the heterogeneous sequence composition patterns of lncRNAs.

## Material and Methods

### Selection of human PRC2-positive and PRC2-negative lncRNAs

Khalil *et al*. used RIP-chip experiments to examine the interaction between ~900 human lncRNAs and SUZ12 or EZH2, two well-known core subunits of PRC2, in three human cell types: HeLa, lung fibroblasts and foot fibroblasts^9^. In these lncRNAs, 261 were found to have physical interactions with PRC2 in at least one cell type, which are defined as human PRC2-positive lncRNAs here. Besides, 227 lncRNAs that are expressed in these cells but failed to show detectable interaction with PRC2 are defined as human PRC2-negative lncRNAs.

To infer the transcription start site (TSS) and then the coding strand of each lncRNA, we collected all available ChIP-Seq data of histone mark H3K4me3, which is known to be mainly associated with active gene promoters, from ENCODE project^40^,^41^. All ChIP-Seq reads were mapped to both ends of each annotated lncRNA locus and the one with higher overall H3K4me3 signal intensities was considered as putative TSS, leaving the other end as putative transcription end site (TES). To assess the validity of this approach, we applied it to RefSeq annotated protein-coding genes^42^ and found for the 15655 genes longer than 5 kb, the accuracy is around 86%. Additionally, since many lncRNAs used in this study lack reliable exon annotations, we use the whole gene body of lncRNAs to perform sequence composition analysis (a detailed explanation can be found in Supplementary text).

### Decomposition of long DNA sequences into transitions between adjacent nucleotides and selection of differentially favored transitions as sequence features

To start sequence decomposition, each sequence is considered as Markov chain of transitions between neighboring nucleotides. Then, the composition of this sequence can be described by the frequencies of observing all possible transitions in it, which are produced by enumerating the order of Markov chain from 0 to m (here m = 5 was used, resulting in a total number of 5460 different transitions). Taking order-4 transition CATG→A as an example (Fig. 1B), its transition frequency in a given sequence is calculated as


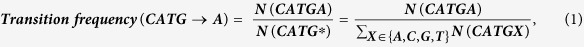


Here, *N(CATGA)* is the times of observing 5-mer CATGA in this sequence.

To find transitions differentially favored by the sequences of PRC2-positive and PRC2-negative lncRNAs as sequence features of PRC2-binding lncRNAs, Welch’s two-sample t-test is applied to compare the frequencies of each transition between these two sequence groups. If the frequencies of a transition in PRC2-positive lncRNAs are significantly higher (lower) than those in PRC2-negative ones with P-value < 0.05, it will be classified as a PRC2-favored (disfavored) transition.

### Examine the distribution of PRC2-favored and disfavored transitions on the quad-tree

The distribution of human PRC2-favored and disfavored transitions on the quad-tree was examined from two perspectives. First, the number of favored and disfavored transitions on each level was counted and compared with that got from 1000 sets of randomized PRC2-positive and PRC2-negative lncRNAs. In each randomized lncRNA set, the original group label of 488 human PRC2-positive and PRC2-negative lncRNAs were randomly shuffled, and transitions differentially favored by these randomized lncRNAs were re-selected using the same criteria. Finally, an empirical *P*-value was calculated for each level as the fraction of randomized lncRNA sets that resulted in an equal or higher number of PRC2-favored/disfavored transitions on this level.

To check whether the PRC2-favored/disfavored transitions prefer to connect with each other and form consecutively favored/disfavored paths (CFPs/CDPs), the fraction of them located in CFPs/CDPs was calculated and compared to that got from 1E + 06 times of random permutations. In each random permutation, all PRC2-favored/disfavored transitions were randomly re-selected from the tree, with keeping the number of selected transitions on each level unchanged. Then, an empirical *P*-value was calculated as the fraction of random permutations that led to an equal or higher proportion of PRC2-favored/disfavored transitions falling in CFPs/CDPs.

### Prediction of PRC2-binding lncRNAs based on the sequence composition of lncRNAs

To build the prediction model of PRC2-positive lncRNAs, the frequencies of all PRC2-favored and disfavored transitions/K-mers were used as predictors, and Bayesian additive regression trees (BART)^21^ analysis was applied to perform model fitting. Here BART was called by using its R package implementation with the default parameter settings, except the number of regression trees was set to be 500. The overall performance of each model was quantified by the area under the receiver operating characteristic (ROC) curve (AUC), which equals 1 if the model made a perfect prediction and 0.5 if the prediction was random. Besides, a more stringent approach of model building, termed as fully blind method, was also used here. The only difference of this approach is that predictor selection was repeatedly performed at each cross-validation step and only lncRNAs in the training set can be used to identify transitions differentially favored by PRC2-positive and PRC2-negative lncRNAs as predictors. In the cross-species prediction, no cross-validation was performed and all human PRC2-positive and PRC2-negative lncRNAs were used to train the prediction model, which was then applied to mouse PRC2-positive and PRC2-negative lncRNAs.

### Definition of PRC2-favored/disfavored fragments and conservation analyses

To find lncRNA fragments that are highly enriched for PRC2-favored transitions and depleted of PRC2-disfavored ones, each human PRC2-positive lncRNA was scanned by a sliding window of size 500 bp, and a local consistency score was assigned to the DNA sequence in the window, which is defined as the sum of the frequencies of all PRC2-favored transition in this sequence fragment minus the sum of the frequencies of all PRC2-disfavored ones. Then, the 500-bp fragment with the highest/lowest score of each PRC2-positive lncRNA was defined as its PRC2-favored/disfavored fragment, respectively.

To measure the conservation levels of these fragments, PhastCons conservation scores of human genome were downloaded from UCSC genome browser (the 44-way version was used here)^43^. Additionally, 1354034 conserved elements annotated by GERP (Genomic Evolutionary Rate Profiling) software were obtained from its website^25^,^26^, which cover about 7% of the human genome. To assess whether the selected lncRNA fragments contain more conserved elements than expected by chance, 1E + 05 times of random simulations were performed. At each time, a 500-bp fragment was randomly chosen from each PRC2-positive lncRNA, and the fraction of these random fragments that overlap with the conserved elements was calculated. Finally, an empirical *P* value was calculated as the proportion of simulations showing an equal or higher fraction of overlapping.

## Additional Information

**How to cite this article**: Tu, S. *et al*. The PRC2-binding long non-coding RNAs in human and mouse genomes are associated with predictive sequence features. *Sci. Rep.* **7**, 41669; doi: 10.1038/srep41669 (2017).

**Publisher's note:** Springer Nature remains neutral with regard to jurisdictional claims in published maps and institutional affiliations.

## Supplementary Material

Supplementary Text and Figures

Supplementary Dataset 1

Supplementary Dataset 2

Supplementary Dataset 3

Supplementary Dataset 4

Supplementary Dataset 5

Supplementary Dataset 6

Supplementary Dataset 7

## Figures and Tables

**Table 1:** Distribution of human PRC2-favored transitions on each level of the quad-tree.

Level	Count	[Q1, Q3]	*P* value
1	0	[0, 0]	1
2	0	[0, 0]	1
3	0	[0, 1]	1
4	3	[2, 7]	0.63
5	34	[16, 30]	0.19
6	203	[87, 115]	0.007
All	240	[108, 151]	0.048

Here the count refers to the number of PRC2-favored transitions on each level. Q1 and Q3 corresponds to the first and third quartile of this count obtained from 1000 randomized lncRNA sets, respectively. The *P* value associated with each level was calculated as the fraction of randomized lncRNA sets having an equal or larger number of PRC2-favored transitions on this level than that observed from real data.
