# Supplementary material for: The PRC2-binding long non-coding RNAs in human and mouse genomes are associated with predictive sequence features
Source: Sci Rep. 2017 Jan 31;7:41669. doi: 10.1038/srep41669 (PMC5282597; doi:10.1038/srep41669)
Supplement: Supplementary Text and Figures [file srep41669-s1.pdf]

The PRC2-binding long non-coding RNAs in human and mouse genomes are associated with predictive sequence features

Shiqi Tu[1, 2], Guo-Cheng Yuan[3, 4, 5], Zhen Shao[1,*]

[1]Chinese Academy of Sciences Key Laboratory of Computational Biology, Collaborative Innovation Center for Genetics and Developmental Biology, Chinese Academy of Sciences-Max Planck Society Partner Institute for Computational Biology, Shanghai Institutes for Biological Sciences, Chinese Academy of Sciences, Shanghai 200031, China.

[2]Graduate University of Chinese Academy of Sciences, Beijing 100049, China

[3]Department of Biostatistics and Computational Biology, Dana-Farber Cancer Institute, Boston, MA 02215, USA.

[4]Department of Biostatistics, Harvard T.H. Chan School of Public Health, Boston, MA 02115, USA.

[5]Harvard Stem Cell Institute, Cambridge, MA 02138, USA

Supplementary text

Analysis of the sequence features associated with transcription factor CTCF's DNA binding

To dissect whether the sequence features identified by our sequence composition analysis pipeline, as well as the consecutively favored paths (CFPs) formed by them with significant preference, have any connection with the sequence specificity of protein binding, we applied our pipeline to analyze the DNA sequences bound by transcription factor (TF) CTCF in human H1 embryonic stem cells. We applied MACS to the ChIP-Seq data of CTCF in H1 cell line generated by ENCODE project[1] , and picked 1000 top CTCF non-promoter binding peaks from MACS output. Then, we extracted the 1-kb long DNA sequences around the summit of these peaks, and defined them as CTCF-positive sequences. Here, we removed CTCF promoter peaks that fall within 1.5kb from the transcription start site of any RefSeq annotated gene, as usually a large fraction of CTCF binding sites in mammalian cells are located in gene promoters[2] and the sequence composition of gene promoters is quite different from the other parts in genome. On the other hand, we randomly selected the same number of 1-kb sequences from the intergenic regions of human genome that don't overlap with any CTCF peak, and defined them as CTCF-negative sequences. Next, we applied the sequence composition analysis pipeline to these two groups of sequences, and identified 558/389 transitions that are significantly favored/disfavored by CTCF-positive sequences compared to the negative ones with P-

value lower than 1E-15 (by 2-tailed Welch t-test). Surprisingly, a very large fraction of CTCF-favored transitions are located in CFPs and, especially, 52 CFPs formed by these transitions are of full length, which means they start from the root of the quad-tree and end at the 6th level (Supplementary Fig. S1C).

A direct by-eye comparison between the DNA binding motif of CTCF obtained from JASPAR database[3] and a representative full-length CFP suggests that the 6-mer formed by all the 6 consecutive transitions on this CFP can be well aligned with a highly informative part of CTCF's binding motif (Supplementary Fig. S1C). To systematically investigate this associations, we defined a motif score for each 6-mer to measure its similarity with the given motif as

$$\textbf{Motif score} = \max_{t \in \{0,1,\ldots,L-6\}} \{\sum_{i=1}^{6} \log_{10}(P(t+i, S_i))\} \quad (1)$$

Here L is the length of motif, *P(i,j)* is the element of its position frequency matrix at row *i* and column *j*, and S_i is the numerical expression of the ith nucleotide of this 6-mer (S_i=1, 2, 3, 4 corresponds to nucleotide A, C, G, T, respectively). Additionally, if a 6-mer's motif score is lower than that of its reverse complementary, the higher score will be assigned to both of them. Then, we divided all the 4^6 possible full-length paths on the quad-tree into 4 groups, based on the length of the longest CFP on each path (here isolated CTCF-favored transitions were taken as CFPs of length 1), and calculated the motif score of the 6-mer corresponding to each full-length path. Interestingly, the CTCF motif scores of these K-mers obviously correlate with the maximum CFP lengths of these paths, and the 52 full-length CFPs have the highest motif sores among the 4 groups (Supplementary Fig. S1D), suggesting the preference of those favored transitions to be connected with each other across adjacent tree levels is not a trivial observation and is intrinsically connected with the sequence specificity mediating protein bindings.

Building empirical classification models of PRC2-binding lncRNAs

To investigate whether PRC2-binding lncRNAs can be predicted by their sequence features in a simple way, as well as to infer the improvement of prediction accuracy achieved by the model fitting process using BART, we additionally devised an empirical classification model to predict human PRC2-binding lncRNAs without involving BART to perform sophisticated model fitting, which is named as "reward-and-punish" model here. We first build this model using the fully blind method as described in main text. Again, all human PRC2-positive and PRC2-negative lncRNAs are divided into 10 subgroups. At each step, one subgroup is selected as the testing set, leaving the other 9 subgroups to serve as the training set, and only lncRNAs in the training set can be used to identify PRC2-favored and disfavored transitions as predictors (here the same cutoff P<0.05 is used). Then, for each lncRNA in the testing set, the frequency of observing each PRC2-favored/disfavored transition in its sequence,

saying $F_{testing}$, is compared with the frequencies of this transition in the sequences of training lncRNAs, and the reward and punishment to this lncRNA are determined based on following rules:

1) For each PRC2-favored transition, check whether $F_{testing}>\mu_{negative}+b*\sigma_{negative}$ is true ($\mu_{negative}$ and $\sigma_{negative}$ is the mean and standard deviation of the frequencies of this transition of PRC2-negative lncRNAs in the training set, respectively, and b is a constant), and give the testing lncRNA a reward 1/M if the answer is yes (M is the number of predictors identified from the training lncRNAs at this step); meanwhile, check whether $F_{testing}<\mu_{positive}-b*\sigma_{positive}$ is true ($\mu_{positive}$ and $\sigma_{positive}$ is the mean and standard deviation of the frequencies of this transition of PRC2-positive lncRNAs in the training set, respectively), and give the lncRNA a punishment 1/M if the answer is yes.

2) For each PRC2-disfavored transition, check whether $F_{testing}<\mu_{negative}-b*\sigma_{negative}$ is true, and give the testing lncRNA a reward 1/M if the answer is yes; meanwhile, check whether $F_{testing}>\mu_{positive}+b*\sigma_{positive}$ is true, and give the lncRNA a punishment 1/M if the answer is yes.

Finally, the prediction score of each testing lncRNA is calculated as the sum of all the reward got by it minus the sum of all the punishment got by it. In this way, we found human PRC2-positive lncRNAs can be better distinguished from PRC2-negative ones using the empirical model with b lying between 0 and 0.5 (Supplementary Fig. S2B), and the highest AUC value was close to 0.60, which is still lower than that got by the prediction model fitted by BART using the same fully blind method. Interestingly, the empirical models with b>1 (which means the testing lncRNAs have low likelihood to get reward and punishment) got AUC values close to or lower than 0.5 (Supplementary Fig. S2B), implying prediction of PRC2-binding lncRNAs based on their sequence composition can be better achieved by combining a number of weak classifiers.

On the other hand, we also tried building an empirical classification model using the non-blind CV process as described in main text. Here, we directly chose the 240 and 87 human PRC2-favored and disfavored transitions identified in main text as predictors, and divided all PRC2-positive and PRC2-nagative lncRNAs into 10 subgroups to perform a 10-fold cross-validation. Next, we adopted the same rules to calculate the reward and punishment for each testing lncRNA. By this means, the empirical model exhibited a similar performance (AUC~0.8-0.85 by models with b lying between 0 and 2, Supplementary Fig. S2C) to that achieved by the prediction model fitted by BART using the non-blind CV method.

Evaluate the *P*-value cutoff used for predictor selection

To see whether the prediction can be made with a smaller number of predictors compared with the original prediction model shown in main text, we tried building a prediction model using only the human PRC2-favored and disfavored transitions with *P*-value<0.01 as predictors. By this means, 66 transitions in total were selected, and the prediction model exhibited a similar (AUC=0.81 using the

non-blind CV method) or reduced accuracy (AUC=0.61 using the fully blind method). Considering the second method is more stringent, we speculate that *P*-value<0.05 can serve as a more reasonable cutoff for predictor selection than *P*-value<0.01. Besides, this finding also implies that the relatively low accuracy of the prediction model built by the fully blind method may not be simply explained by over-fitting, which is usually indicated by an observation that the model using more predictors tends to show a lower accuracy, as evaluated by the testing set[4].

Explanation of using the whole gene body of lncRNAs for sequence composition analysis

In this study, we extracted the sequence of the entire gene body of each lncRNA to perform sequence composition analysis. This is mainly due to two reasons. First, several recent studies published by different labs suggested that EZH2/PRC2 may directly interact with the nascent transcripts of many genes [5-7], and a very large fraction of the interaction sites identified from corresponding CLIP-seq experiments were found to be located in regions annotated as introns[6,8,9]. Second, the lncRNAs used here were initially profiled using custom-design tiling arrays, which are known to have a lower resolution and higher noise levels than sequencing based platforms. For example, we collected the annotation of 4859 lncRNA exons from Khalil *et al.*[10] (in total they cover about 16.24% of the gene body of these lncRNAs), and found quite a number of the 488 human lncRNAs used in this study have no exon annotations (Fig. 2B). Moreover, these exon annotations show clear differences from other gene annotations, such as those provided by RefSeq (Supplementary Fig. S2G-H). On this account, we finally decided to use the entire gene body of lncRNAs to perform sequence composition analysis, in order to reduce the risk of missing regions potentially important for PRC2-lncRNA interactions.

On the other hand, we also found that the difficulty posed by using the entire gene body for sequence analysis can be largely overcome by focusing the study on sequence features associated with PRC2-lncRNA interactions. Here we use the PRC2-favored and disfavored fragments identified by us as examples. 16.9% of the PRC2-disfavored fragments overlap with the exons obtained from Khalil *et al.*, which is close to the fraction of the lncRNA gene bodies covered by these exons, and this fraction for PRC2-favored ones is as high as 26.2%. Of note, we have shown ~30% of the PRC2-favored fragments overlap with annotated conserved elements, indicating a considerable part of the important building blocks of these lncRNAs may not be covered by these 4859 exons. Beside the lncRNA shown in Fig. 2B, we additionally selected two representative human PRC2-binding lncRNAs to illustrate the power of sequence composition analysis. For the well-studied PRC2-binding lncRNA XIST, its PRC2-favored fragment is at the second exon (based on RefSeq gene annotations, Supplementary Fig. S2G), which is not in the exon list obtained from Khalil *et al.* On the other hand, we found the PRC2-favored fragment of lncRNA chr13:90799274-90818300 is not covered by either RefSeq annotated gene exons (the closest RefSeq annotated lncRNA is MIR17HG) or the exons got from Khalil *et al.* (Supplementary Fig. S2H). However, by incorporating a recently published RIP-seq

dataset of EZH2 and SUZ12 in K562 cells, we found the transcript generated from this fragment can strongly interact with these two PRC2 core subunits (Supplementary Fig. S2H), suggesting our analysis can largely recover the missing building blocks important for the function of PRC2-binding lncRNAs.

Definition of mouse PRC2-binding lncRNAs

We collected 1666 putative mouse lncRNAs from Guttman *et al.*[11], which were discovered by using a similar method to the human lncRNAs studied in Khalil *et al.*[10], and lifted-over them to mm9 mouse genome assembly. Next, we obtained 8670 PRC2-associated RNA tags from Zhao *et al.*[12], which were generated by RIP-seq experiments against EZH2 in mouse embryonic stem cells (mESCs), and mapped them to these mouse lncRNAs. In total, we got 283 mouse lncRNAs that overlap with PRC2-associated RNA tags. Meanwhile, we also mapped Pol2 ChIP-Seq peaks of mESCs to these mouse lncRNAs, and found 540 mouse lncRNAs contain at least one Pol2 peak, which were taken as lncRNAs expressed in mESCs[13]. Finally, we defined mouse PRC2-positive lncRNAs as the 153 mouse lncRNAs that overlap with both PRC2-associated RNA tags and Pol2 ChIP-Seq peaks, and defined mouse PRC2-negative lncRNAs as the other 540-153=387 mouse lncRNAs that overlap with Pol2 ChIP-Seq peaks but contain no PRC2-associated RNA tag.

To further validate our cross-species prediction, we collected 13,764 putative RNA-contact sites (RCSs) of EZH2 in mESCs from Kaneko *et al* [6] (by personal communication with the authors. The list of RCSs obtained from the GEO webpage of Kaneko *et al* contains only 6784 RCSs, which was said to be generated using slight different and more stringent parameters), which were identified from the corresponding PAR-CLIP-seq data, and mapped them to the 540 putative mESC-expressed lncRNAs. As a result, 144 lncRNAs were found to contain at least one RCS, and 60 of them (41.7%) were previously classified as mouse PRC2-positive lncRNAs based on the RIP-seq data of EZH2 published in Zhao *et al* [12]. More explicitly, 39.2% of mouse PRC2-positive lncRNAs contain at least one RCS of EZH2, while this fraction for mouse PRC2-negative lncRNAs is only 21.7%. Furthermore, mouse PRC2-positive lncRNAs are also more likely to contain more than one RCSs than PRC2-negative ones (Supplementary Fig. 3D). Interestingly, we found for both mouse PRC2-positive and PRC2-negative lncRNAs, lncRNAs with high prediction scores derived from the model trained with human lncRNAs (Fig. 3D and Supplementary Fig. 3F) and also from the model trained with mouse lncRNAs (Supplementary Fig. 3E) are more likely to contain EZH2 RCS than those with low prediction scores, indicating the definition of PRC2-positive ad PRC2-negative lncRNAs only based on single RIP/CLIP-seq dataset may not be quite reliable and inherent sequence patterns can potentially be utilized to correct the false positives and false negatives in them. Then, we defined the 144 lncRNAs that contain at least one RCS as RCS-containing lncRNAs and the other 540-144=396 lncRNAs as RCS-null ones. By using the prediction model trained with human lncRNAs, we found mouse RCS-containing

lncRNAs can be distinguished from RCS-null ones with considerable accuracy (AUC=0.66, Fig 3E). Finally, to combine these two classes of lncRNA labels, we defined the 60 mouse PRC2-positive lncRNAs that also contain EZH2 RCS as high-confidence mouse PRC2-positive lncRNAs, and the 303 mouse PRC2-negative lncRNAs without any EZH2 RCS as high-confidence mouse PRC2-negative lncRNAs. Again, by using the human prediction model to perform cross-species prediction, we found high-confidence mouse PRC2-positive lncRNAs can be distinguished from the high-confidence PRC2-negative ones with clearly better accuracy (AUC=0.72, Fig 3E).

Compare RNA contact sites of EZH2 with PRC2-favored fragments

Here, we tried using the EZH2 RCSs to assess the method we proposed to recognize PRC2-favored fragments in human lncRNAs. Again, a 500bp sliding window was used to scan each mouse RCS-containing lncRNA, and the local consistency score of the sequence fragment in the sliding window was calculated as the sum of the frequencies of all human PRC2-favored transitions in this sequence fragment minus those of all human PRC2-disfavored ones. Then, we selected the fragment with the highest and the lowest consistency score of each mouse RCS-containing lncRNA as its PRC2-favored and disfavored fragment, respectively. Of note, here we used the sequence features of human PRC2-binding lncRNAs to detect PRC2-favored/disfavored fragments for mouse lncRNAs, so that these mouse fragments were identified by using the same sequence model as the human ones. Interestingly, similar to what we observed in human, 37.5% of mouse PRC2-favored fragments (54 of 144) overlap with mouse conserved elements (also annotated by GERP program), and this fraction is significantly higher than that expected by chance (right-tailed *P*-value<1E-06 by the same random permutation test as that shown in Fig. 2D). Meanwhile, only 10.4% of mouse PRC2-disfavored fragments overlap with conserved elements (15 of 144), which is slightly lower than that expected by chance (left-tailed *P*-value=0.03). Again, these findings can support our hypothesis that a considerable proportion of the sequence patterns associated with in vivo PRC2-lncRNA interactions are shared between human and mouse.

On the other side, we only observed a small number of mouse PRC2-favored fragments (9.7%, 14 of 144) directly cover EZH RCS, though it’s still significantly higher than expected by chance (right-tailed *P*-value=0.0097 by random permutation test) and also higher than that of mouse PRC2-disfavored fragments (4.2%, 6 of 144, left-tailed *P*-value =0.3931). (If we switched to use those 6784 more stringent RCSs, we got 92 RCS-containing lncRNAs. Among these lncRNAs, 11 of the PRC2-favored fragments directly cover EZH2 RCS, with empirical *P*=0.0013, and only 1 PRC2-disfavored fragment overlaps with RCS.) It should be of note that the main purpose of defining PRC2-favored fragments is to investigate whether the aggregation of the sequence features associated with PRC2-lncRNA interactions at certain genomic regions is potentially linked with the functional importance of these regions, and for simplicity, we only took one 500bp fragment with the highest score of each

PRC2-binding lncRNA as the representative region for downstream analysis (here PRC2-disfavored fragment with the lowest score of each lncRNA is used as control). As we have found PRC2-lncRNA interactions in human and mouse exhibit clear sequence specificities, it's reasonable to speculate the direct contact sites of PRC2 on lncRNAs are very likely to be enriched with sequence features associated with PRC2-lncRNA interactions, but we do not intend to say PRC2-lncRNA interactions should always happen at the 500bp fragment with the highest score of each lncRNA. Following this direction, we calculated the local consistency score around the 400 EZH2 RCSs falling in the 540 mouse lncRNAs used in this study, which was defined to the 500bp sequence fragment centered at the middle of each RCS. Besides, we generated 100 sets of random control regions for these RCSs and at each time a 500bp control region was randomly selected for each RCS from the same lncRNA without overlapping with any identified EZH2 RCS. Interestingly, the 400 EZH2 RCSs falling in lncRNAs in general exhibit obviously higher local consistency scores than the random control regions (Supplementary Fig. 3G), suggesting the local sequences around these RCSs are more enriched with the sequence features associated with PRC2-lncRNA interactions compared to the other parts of the lncRNAs they belong to.

Sequence feature analysis of the RNA contact sites of EZH2 falling in lncRNAs

To directly find the sequence features associated with the RNA contact sites of PRC2 on lncRNAs, we specially analyzed the composition of sequences surrounding the EZH2 RNA contact sites (RCSs) identified from PAR-CLIP-seq data in mESCs. First, EZH2 RCSs located within 500bp from each other were merged together, and we defined EZH2 RCS fragment as the 500bp sequence fragment surrounding the center of each merged RCS. Since this study mainly focuses on interactions between PRC2 and lncRNAs, we only took the 310 RCS fragments falling in the 540 putative ESC-expressed lncRNAs for sequence analysis. As negative controls, we randomly chose the same number of 500bp sequence fragments from the high-confidence mouse PRC2-negative lncRNAs defined by us. By using our sequence composition analysis pipeline to compare the 310 EZH2 RCS fragments with the negative control sequences, we identified 190/110 transitions that are significantly favored/disfavored by EZH2 RCS fragments (using P-value<0.05 as cutoff). However, this time we found thymine (T) is significantly over-represented in these fragments and cytosine (C) is significantly under-represented (Supplementary Table 7). Specifically, for those transition of order 0-2, all the transitions that are significantly favored by RCS fragments end up with T, and all the transitions are significantly disfavored end up with C, though not all the transitions that end up with T/C are significantly favored/disfavored. As these RCSs were originally detected by taking advantage of the T-to-C transitions produced by PAR-CLIP procedure[6,14], we speculate the enrichment of T and depletion of C in EZH2 RCSs may not be real sequence features of PRC2's RNA contact, and moved to rescale the frequencies of all the transitions of order>0 by the frequency to observe their last nucleotide in each sequence (the 4 order-0 transitions, i.e. the nucleotide frequencies of A,T,G and C, were

excluded from the following analysis). For example, rescaled transition frequency CATG→A of a sequence is calculated as the original transition frequency CATG→A divided by the frequency of observing nucleotide A in this sequence. After rescaling, we repeated the feature selection procedure, and got 181/99 transitions that are significantly favored/disfavored by EZH2 RCS fragments, which were name as RCS-favored and disfavored transitions (Supplementary Table 7).

Again, we used a complete quad-tree of height 6 to visualize the distribution of these selected transitions (Supplementary Fig. 4A). The RCS-favored transitions were also found to strongly prefer to form consecutively favored paths (CFPs, Supplementary Fig. 4B), while the RCS-disfavored ones showed a relatively weak preference to form consecutively disfavored paths (CDPs, Supplementary Fig. 4C). Finally, we applied the same fully blind approach and built a prediction model to distinguish the 310 EZH2 RCS fragments falling in lncRNAs from the corresponding negative control sequences. In this way, the model achieved a clearly lower accuracy (AUC=0.59, Supplementary Fig. 4C) compared to the prediction of RCS-containing lncRNAs.

In addition, we also performed de novo motif discovery around the RNA contact sites of EZH2. Again, we took the 310 500bp EZH2 RCS fragments falling in lncRNAs as well as the corresponding negative control sequences selected from high-confidence mouse PRC2-negative lncRNAs. Then, we randomly split both the RCS fragments and the negative control sequences into two subgroups of equal size, and only used the first subgroup as input for the MEME suite (both MEME and DREME in the suite were used here) to perform de novo motif finding[15]. MEME was ran with the default parameter setting, and the analysis with DREME was carried for two times. In the first time, we used the first subgroup of negative control sequences as input control sequences, and in the second time, we asked DREME to create a set of control sequences by randomly shuffling the RCS sequences while preserving their dimer frequencies. Finally, all the 78 motifs detected by MEME and DREME were collected and applied to the second subgroup of RCS fragments and the corresponding negative control sequences to perform motif enrichment analysis[16]. For each candidate motif, we compared the fraction of RCS fragments that contain this motif to that of the corresponding negative control sequences, and used Fisher's exact test to check whether this motif is significantly over-represented in the RCS fragments compared to the negative control sequences (the first column of *P*-values shown in Supplementary Table 8). Besides, we generated another set of random sequences by shuffling the sequences of RCS fragments, as they have been found to contain significantly more thymine (T) and less cytosine (C) than the negative control sequences, and used them as controls to perform a second motif enrichment test with the RCS fragments (the second column of *P*-values shown in Supplementary Table 8). Unfortunately, none of the 78 motifs obtained from de novo motif finding showed significant enrichment in both of the two tests. This finding indicates it could be quite difficult to find the RNA sequence motifs that can be directly recognized by EZH2, which is consistent

with the hypothesis suggested by several recent studies that the mechanisms governing PRC2's RNA binding may be very complicated[17,18].

Decomposition of long sequences into K-mers and prediction of PRC2-binding lncRNAs based on K-mer frequencies

To compare with transition based sequence decomposition, traditional K-mer based method was also applied to analyze the sequence composition of human lncRNAs. In this approach, the composition of a sequence is described by the frequencies of observing all the possible K-mers of length from 1 to m+1 (here m=5, resulting in a total number of 5460 different K-mers, which can also be visualized by a complete quad-tree of height 6). Taking the 5-mer CATGA as an example, the corresponding K-mer frequency was defined as the frequency of observing it in the given sequence

$$\mathbf{K-mer\ frequency\ (CATGA)} = \frac{\boldsymbol{N(CATGA)}}{\boldsymbol{N-K+1}} \qquad (2)$$

Here, *N(CATGA)* is the times of observing 5-mer CATGA in this sequence, and N is the length of this sequence. Finally, K-mers significantly under- or over-represented in the sequences of PRC2-positive lincRNAs compared to PRC2-negative ones were identified by using the same statistical test and *P*-value cutoff as that used for transition frequencies.

In main text, we have shown the prediction models based on transition frequencies using the fully blind method showed superior accuracies in predicting both human and mouse PRC2-binding lncRNAs (Fig. 4A-B), especially for the extremely long lncRNAs. Additionally, we also checked the performance of the prediction models built by the non-blind CV method on the moderately long and extremely long subgroup of lncRNAs separately, and again got similar results (Supplementary Fig. S5A-B), indicating this finding is not specific to the method we chose to build the prediction model. Moreover, to control the impact of model complexity, we chose to build prediction models using a fixed number of top K-mer or transition frequencies (ranked by the P-value of Welch's t-test) as predictors at each time. Still, a consistent improvement of prediction accuracy on the extremely long subgroup of human lncRNAs was observed for the models based on transition frequencies, as compared to the K-mer based models using the same number of predictors (Supplementary Fig. S5C, here the prediction models were built by the non-blind CV method).

In the following part, we use a highly idealized theoretical model to illustrate the difference between transition and K-mer based feature selections. Suppose a 6-mer, saying CAGTCT, is the sequence feature mediating the interactions between a protein and DNA/RNA sequences. In positive sequences, the frequency of observing this K-mer can be expected as

$$\boldsymbol{Frequency_{positive}^{K-mer}} = \frac{\boldsymbol{M + N\prod_{i=1}^{6} P(S_i)}}{\boldsymbol{N}} = \prod_{\boldsymbol{i=1}}^{\boldsymbol{6}} \boldsymbol{P(S_i)} * \left(\boldsymbol{1} + \frac{\boldsymbol{M}}{\boldsymbol{N\prod_{i=1}^{6} P(S_i)}}\right) \qquad (3)$$

. Here we ignored the difference between N and $N-K+1$ as typically $K<<N$, and $P(S_i)$ is the frequency of observing the i-th nucleotide of this 6-mer, and M stands for the extra number of occurrences of the 6-mer in each positive sequence as needed for the interaction, which is set to be 0 for negative sequences. Thus, its frequency in negative sequences can be expected as

$$Frequency_{negative}^{K-mer} = \frac{N\prod_{i=1}^{6}P(S_i)}{N} = \prod_{i=1}^{6}P(S_i) \quad (4)$$

. For the transition-based method, we use the last transition $CGCGC \rightarrow A$ of this 6-mer as an example. Its frequency in positive sequences can be expected as

$$Frequency_{positive}^{Transition} = \frac{M + N\prod_{i=1}^{6}P(S_i)}{M + N\prod_{i=1}^{5}P(S_i)} = P(S_6) * \left(1 + \frac{M(1 - P(S_6))}{M * P(S_6) + N\prod_{i=1}^{6}P(S_i)}\right) \quad (5)$$

, and its frequency in negative sequences can be expected as

$$Frequency_{negative}^{Transition} = \frac{N\prod_{i=1}^{6}P(S_i)}{N\prod_{i=1}^{5}P(S_i)} = P(S_6) \quad (6)$$

. For both of the K-mer and the transition based methods, it's easy to find the frequencies in positive and negative sequences can be expressed as C*(1+Δ) and C*1, respectively. Here C is a constant between two sequence groups. Moreover, we simplify the nucleotide frequencies of A, C, G and T as 1/4. Then, $\Delta_{\text{k-mer}}$ and $\Delta_{\text{transition}}$ can be expressed as

$$\Delta_{K-mer} = \frac{M * 4^6}{N} \quad (7)$$

and

$$\Delta_{Transition} = \frac{M * 4^6}{(N + M * 4^5)} * \frac{3}{4} \quad (8)$$

This idealized model suggests, when N is a constant for all sequences, the difference in K-mer frequencies between positive and negative sequences should be greater than that in transition frequencies.

For our analysis, however, the problem is that N is quite large and varies dramatically across different lncRNAs (10^0-10^2 Kb). Specifically, we put the length of 261 human PRC2-binding lncRNAs into above formulas and used one-sample Students' t-test to separately compare $\Delta_{\text{k-mer}}$ and $\Delta_{\text{transition}}$ with zero (here M is set to be 1 to mimic the most extreme situation). Interestingly, the t-statistics for $\Delta_{\text{k-mer}}$ and $\Delta_{\text{transition}}$ equals to 16.9 and 23.7, respectively, which is opposite from that expected for the case with constant N. Besides, protein-DNA interactions usually happen on small sequence fragments of length 10-30 base pairs, and they are often associated with clear sequence specificities[3].

However, the interaction between a protein complex and a long RNA molecular may take place at multiple loci on this RNA transcript[6], as they usually have highly complicated high-order structures, and the sequence features associated with such interactions might be quite diffusive on lncRNAs, which suggests the sequence composition analysis based on transition frequencies can also be a plausible way for lncRNAs.

Prediction of human PRC2-binding lncRNAs based on the composition of sequences near the transcription start site of lncRNA genes

It has been revealed that PRC2 may preferentially interact with lncRNAs at regions close to their 5' ends[19,20]. Inspired by these findings, we specifically investigated the sequences close to the transcription start site (TSS) of human PRC2-binding lncRNAs. First, we define the TSS region of each lncRNA as the region spanning from the TSS to 1kb downstream of the TSS (Supplementary Fig. S6A). Then, we applied our sequence composition analysis pipeline to compare the sequences extracted from the TSS region of human PRC2-positive and PRC2-negative lncRNAs, and identified transitions differentially favored by their TSS regions using the same criteria as the analysis for the whole gene bodies, which were then used to build a prediction model of PRC2-binding lncRNAs using the fully blind method. In this way, the prediction model using transitions with P-value<0.05 as predictors showed a clearly reduced accuracy (AUC=0.57, typically ~250 predictors are used at each cross-validation step), compared with the model using only transitions with P-value<0.01 as predictors (AUC=0.61, typically ~60 predictors are used), indicating the former one may suffer from overfitting[4]. As a comparison, we also defined the transcription end site (TES) region of each lncRNA as the region spanning from 1kb upstream of the TES to the TES (Supplementary Fig. S6A), and applied the same analysis to the sequences extracted from the TES region of PRC2-positive and PRC2-negative lncRNAs. Interestingly, the prediction model using 56 transitions differentially favored by their TES regions (with P-value<0.01) as predictors achieved a slightly lower accuracy (AUC=0.59) than the TSS region based model, implying the sequence composition close to the TSS of each lncRNA may be more predictive of whether this lncRNA can potentially interact with PRC2 than the other regions. To test this hypothesis, we randomly selected 1000 relative positions from [0.1, 0.9] (here the relative position 0 and 1 represent the TSS and TES of each lncRNA, respectively, and the relative positions out of [0.1, 0.9] were excluded to make the selected positions far enough from TES and TSS). For each of these selected positions, we extracted the 1kb sequence around the corresponding position of each human lncRNA, and built a prediction model based on the composition of these sequences to predict whether the sequence is from a PRC2-positive or negative lncRNA. Interestingly, none of the prediction models based on these 1000 randomly selected positions achieved a better accuracy than that got by the TSS region based prediction model, while 29 of them achieved higher AUC values than that got by the TES region based prediction model (Supplementary Fig. S6B). On the other hand, quite a number of these prediction models selected more sequence features as predictors than the

TSS region based model (Supplementary Fig. S6C). This finding suggests the sequence close to the TSS of lncRNAs may be better used to predict whether this lncRNA can potentially interact with PRC2 than the other parts. Recent studies have suggested that some lncRNAs can interact with multiple chromatin modifying complexes at different regions, implying a potential role of these lncRNAs to serve as molecular scaffold to link different functional modules together[20,21]. Then, it's reasonable to speculate that different parts of these lncRNAs may be associated with different functions and, thus, have different sequence patterns. However, due to the resolution of the lncRNA annotations used in this study, it's still difficult to reliably address this question.

Supplementary Figure Legends

Supplementary Figure S1. Consecutively disfavored paths (CDPs) formed by human PRC2-disfavored transitions and the sequence features associated with transcription factor CTCF's DNA binding. (A) A branch of the complete quad-tree that starts from level 4 and contains a CDP of length 2: CCAC➔C➔A. (B) Summary statistics of the CDPs observed in Fig. 1D, which indicate the human PRC2-disfavored transitions have relatively weak preference to connect with each other and form CDPs. (C) A representative full-length CFP formed by 6 consecutive transitions that are significantly favored by the CTCF-binding DNA sequences identified from human H1 cells compared to the sequences not bound by it. These 6 consecutive transitions in together form a 6-mer CCCCCT that can be well matched with the binding motif of CTCF obtained from JASPAR database. (D) Box plot of the motif scores of all the possible full-length paths extracted from the quad-tree, which start from the root and end at each level-6 node. Here the motif score of a path was defined to measure the similarity between CTCF's binding motif and the K-mer formed by all the 6 consecutive transitions on this path, and the full-length paths are grouped by the length of the longest CFP found on them.

Supplementary Figure S2. Analysis of the PRC2-favored and disfavored fragments identified from human PRC2-binding lncRNAs. (A) AUC value of each of the 10 lncRNA subgroups as the testing set. The prediction scores were derived from the prediction model build by the non-blind CV (red stars) or the fully blind (blue stars) method. (B-C) AUC value achieved by the empirical classification model build by the fully blind (B) or the non-blind CV (C) method with different b. (D) Boxplot of EZH2 and SUZ12 fRIP-seq signal intensities at PRC2-favored and disfavored fragments. Here the signal intensity of each fragment was measured as the number of reads mapped to it per million total reads (RPM) and then averaged over 2 (for EZH2) or 3 (for SUZ12) biological replicates. (E) Distribution of the P-values got by using pairwise Students' t-test to compare the average PhastCons conservation scores between the PRC2-favored and disfavored fragments identified from each of the 1000 sets of randomized PRC2-positive lncRNAs. Here 1.7% of the randomized lncRNA

sets achieved *P*-values lower than 1.7E-04, which is got by using the same test to compare the average conservation scores between the PRC2-favored and disfavored fragments identified from the original human PRC2-positive lncRNAs as shown in Fig. 2C, and this fraction is used as an empirical estimate of the false positive rate (FPR) for the test shown in Fig. 2C. (F) List of three tests performed in Fig. 2C-D, together with their empirical P-values and FPR values, which were estimated by using the same method as shown in (E). (G-H) Two representative PRC2-positive lncRNA loci, including the well-known PRC2-binding lncRNA XIST (G). Their PRC2-favored and disfavored fragments are indicated by the red and blue bars, respectively. (I-J) Distribution of PRC2-favored (I) and disfavored (J) fragments in human PRC2-positive lncRNAs. Here relative position 0 and 1 correspond to the 5' and 3' end of each lncRNA, respectively.

Supplementary Figure S3. Overlap between human and mouse PRC2-favored and disfavored transitions. (A) Venn diagram to show the overlap between human and mouse PRC2-favored and disfavored transitions. (B) Scatter plot of the prediction score of each mouse lncRNA obtained from the mouse prediction model trained with mouse lncRNAs using the fully blind method and the prediction score got from cross-species prediction using the human prediction model trained with human lncRNAs. (C) AUC values of three human prediction models in predicting mouse PRC2-binding lncRNAs. Here the models were trained with human lncRNAs using different groups of transitions as predictors, and then applied to mouse lncRNAs. (D) Fraction of mouse PRC2-positive and PRC2-negative lncRNAs that contain exactly one and more than one RCSs of EZH2 identified from the PAR-CLIP-seq data. (E) Fraction of mouse PRC2-positive and PRC2-negative lncRNAs that contain EZH2 RCSs. Here each group of lncRNAs are split into two subgroups of equal size by the median of their prediction scores derived from the prediction model trained with mouse PRC2-positive and PRC2-negative lncRNAs using the fully blind method, and the P-values were calculated by right-tailed Fisher's exact test to measure whether the subgroup of lncRNAs with high prediction scores are significantly more likely to contain EZH2 RCS compared to the subgroup with low prediction scores. (F) The same analysis as that shown in Fig. 3D. Here we switched to use the 6784 RCSs of EZH2 obtained from the GEO webpage of Kaneko *et al*, which were suggested to be generated with more stringent parameters. (G) Cumulative distribution of the local consistency scores of 400 EZH2 RCSs falling in the 540 mouse lncRNAs used in this study (red curve). Here the local consistency score of each RCS was defined as the sum of frequencies of all human PRC2-favored transitions in the 500bp sequence fragment centered at the middle of this RCS minus those of all human PRC2-disfavored ones in this sequence fragment, and each blue dash line represents the cumulative distribution of the local consistency scores of the corresponding 500bp random control regions selected from the same lncRNAs. In total 100 sets of random control regions were generated.

Supplementary Figure S4. Sequence features of EZH2 RNA contact sites (RCSs) falling in lncRNAs. (A) The complete quad-tree of height 6 constituted by all possible transitions of order 0-5 (placed on level 1-6 accordingly). Left panel shows a building block of the quad-tree, which comprises of 4 transitions with the same prefix. Each line represents a transition and the color indicates whether the transition is significantly favored or disfavored by the sequences surrounding the EZH2 RCSs falling in lncRNAs. (B) Summary statistics of the CFPs observed from the quad-tree shown in (A). The empirical *P*-value is estimated by permutation test and suggests the transitions that are significantly favored by EZH2 RCSs strongly prefer to connect with each other and form CFPs. (C) Summary statistics of the CDPs observed from the quad-tree shown in (A). The *P*-value suggests the transitions that are significantly disfavored by EZH2 RCSs have a weak preference form CDPs. (D) ROC curve and corresponding AUC value of the prediction model built by the fully blind method in predicting EZH2 RCS fragments falling in lncRNAs.

Supplementary Figure S5. Compare the performance of prediction models based on K-mer and transition frequencies. (A-B) AUC value of the prediction models based on transition (red bars) or K-mer (blue bars) frequencies, which were trained and tested by the human (A) and mouse (B) lncRNAs, respectively. Here the prediction models were built by the non-blind CV method, and all the human/mouse PRC2-positive and PRC2-negative lncRNA were further divided into two subgroups of equal size by their length, termed as the moderately long and the extremely long subgroup, to access the accuracy of these models on lncRNAs of different length. (C) AUC value of the human prediction models on the moderately long and the extremely long subgroup of human lncRNAs, respectively. Here the models were built by the non-blind CV method, using a fixed number of top transition or K-mer frequencies (ranked by the P-value of Welch's t-test) as predictors. (D) A branch of K-mers cut from the quad-tree, which is constituted by all the possible K-mers of length 1-6, from the same position as the branch shown in Supplementary Figure 1C. Here the edge color indicates whether the K-mer is significantly over-represented (red) or under-represented (green) in CTCF-binding DNA sequences compared to the non-binding sequences.

Supplementary Figure S6. Prediction of human PRC2-binding lncRNAs based on the sequence in the TSS and TES region of each lncRNA. (A) Definition of the TSS and TES region of each lncRNA. (B) AUC values of the prediction models based on the sequences at the TSS/TES region of human lncRNAs (red/blue stars, respectively), as well as the prediction models based on sequences extracted from 1000 randomly chosen positions of the lncRNAs (blue dots shown in the middle), which are used to estimate the empirical *P*-values for the performance of the TSS and TES region based prediction model. (C) Number of sequence features identified from the sequences of TSS and TES

regions (red/blue stars, respectively), as well as the sequences extracted from 1000 randomly chosen positions of the lncRNAs (blue dots shown in the middle). All the sequence features are selected by using the same method and *P*-value cutoff (0.01).

Supplementary Table S1. Distribution of human PRC2-disfavored transitions on each level of the quad-tree.

Level	Count	[Q1, Q3]	*P* value
1	0	[0, 0]	1
2	0	[0, 0]	1
3	1	[0, 1]	0.40
4	4	[2, 7]	0.49
5	14	[16, 29]	0.86
6	68	[84, 111]	0.97
All	87	[103, 146]	0.94

Supplementary Table S2. Summary statistics of using two-sample Welch t-test to compare the frequencies of each transition between human PRC2-positive and PRC2-negative lncRNAs.

Supplementary Table S3. List of the human lncRNAs used in this study and their prediction scores obtained from the prediction models built by the non-blind CV and the fully blind method (based on hg18 genome assembly).

Supplementary Table S4. List of the PRC2-favored and disfavored fragment of each human PRC2-binding lncRNA (based on hg18 genome assembly).

Supplementary Table S5. Summary statistics of using two-sample Welch t-test to compare the frequencies of each transition between mouse PRC2-positive and PRC2-negative lncRNAs.

Supplementary Table S6. List of the mouse lncRNAs used in this study and their prediction scores obtained from the prediction models built by the non-blind CV and the fully blind method (based on mm9 genome assembly).

Supplementary Table S7. Summary statistics of using two-sample Welch t-test to compare the frequencies of each transition between the EZH2 RCS fragments falling in lncRNAs and the corresponding negative control sequences.

Supplementary Table S8. List of motifs detected by de novo motif discovery from the EZH2 RCS fragments as well as their relative enrichment in EZH2 RCS fragments compared to two sets of control sequences generated by different methods.

1 Zhang, Y. *et al.* Model-based analysis of ChIP-Seq (MACS). *Genome Biol* **9**, R137, (2008).

2 Kim, T. H. *et al.* Analysis of the vertebrate insulator protein CTCF-binding sites in the human genome. *Cell* **128**, 1231-1245, (2007).

3 Sandelin, A., Alkema, W., Engstrom, P., Wasserman, W. W. & Lenhard, B. JASPAR: an open-access database for eukaryotic transcription factor binding profiles. *Nucleic Acids Res* **32**, D91-94, (2004).

4 Yuan, Y., Guo, L., Shen, L. & Liu, J. S. Predicting gene expression from sequence: a reexamination. *PLoS Comput Biol* **3**, e243, (2007).

5 Kaneko, S., Son, J., Bonasio, R., Shen, S. S. & Reinberg, D. Nascent RNA interaction keeps PRC2 activity poised and in check. *Genes Dev* **28**, 1983-1988, (2014).

6 Kaneko, S., Son, J., Shen, S. S., Reinberg, D. & Bonasio, R. PRC2 binds active promoters and contacts nascent RNAs in embryonic stem cells. *Nat Struct Mol Biol* **20**, 1258-1264, (2013).

7 Kanhere, A. *et al.* Short RNAs are transcribed from repressed polycomb target genes and interact with polycomb repressive complex-2. *Mol Cell* **38**, 675-688, (2010).

8 Guil, S. *et al.* Intronic RNAs mediate EZH2 regulation of epigenetic targets. *Nat Struct Mol Biol* **19**, 664-670, (2012).

9 Kaneko, S. *et al.* Interactions between JARID2 and noncoding RNAs regulate PRC2 recruitment to chromatin. *Mol Cell* **53**, 290-300, (2014).

10 Khalil, A. M. *et al.* Many human large intergenic noncoding RNAs associate with chromatin-modifying complexes and affect gene expression. *Proc Natl Acad Sci U S A* **106**, 11667-11672, (2009).

11 Guttman, M. *et al.* Chromatin signature reveals over a thousand highly conserved large non-coding RNAs in mammals. *Nature* **458**, 223-227, (2009).

12 Zhao, J. *et al.* Genome-wide identification of polycomb-associated RNAs by RIP-seq. *Mol Cell* **40**, 939-953, (2010).

13 Seila, A. C. *et al.* Divergent transcription from active promoters. *Science* **322**, 1849-1851, (2008).

14 Corcoran, D. L. *et al.* PARalyzer: definition of RNA binding sites from PAR-CLIP short-read sequence data. *Genome Biol* **12**, R79, (2011).

15 Bailey, T. L. *et al.* MEME SUITE: tools for motif discovery and searching. *Nucleic Acids Res* **37**, W202-208, (2009).

16 Xu, J. *et al.* Combinatorial assembly of developmental stage-specific enhancers controls gene expression programs during human erythropoiesis. *Dev Cell* **23**, 796-811, (2012).

17 Betancur, J. G. & Tomari, Y. Cryptic RNA-binding by PRC2 components EZH2 and SUZ12. *RNA Biol* **12**, 959-965, (2015).

18 Cifuentes-Rojas, C., Hernandez, A. J., Sarma, K. & Lee, J. T. Regulatory interactions between RNA and polycomb repressive complex 2. *Mol Cell* **55**, 171-185, (2014).

19 Zhao, J., Sun, B. K., Erwin, J. A., Song, J. J. & Lee, J. T. Polycomb proteins targeted by a short repeat RNA to the mouse X chromosome. *Science* **322**, 750-756, (2008).

20 Tsai, M. C. *et al.* Long noncoding RNA as modular scaffold of histone modification complexes. *Science* **329**, 689-693, (2010).

21 Mercer, T. R. & Mattick, J. S. Structure and function of long noncoding RNAs in epigenetic regulation. *Nat Struct Mol Biol* **20**, 300-307, (2013).

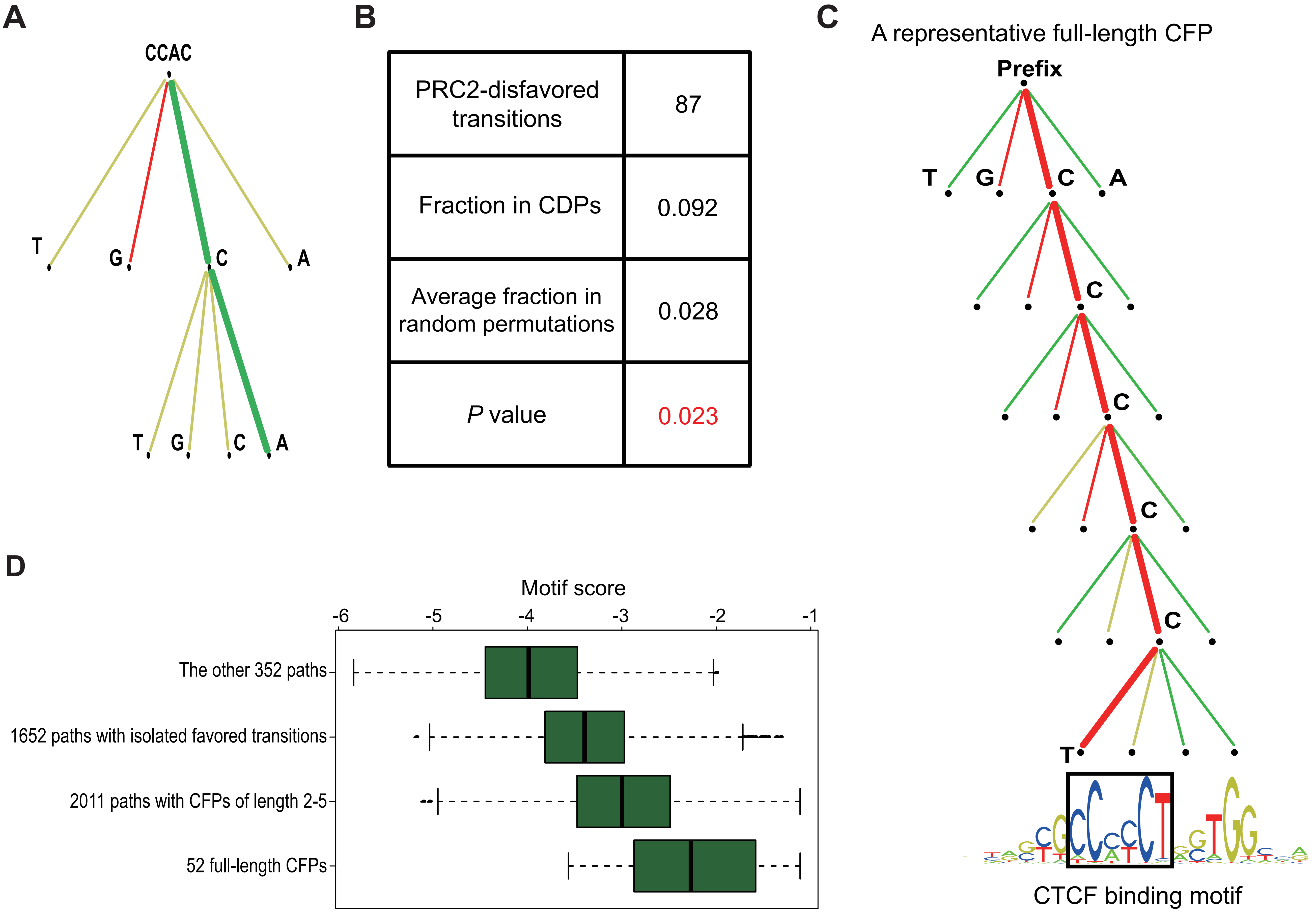

Supplementary Figure S1.

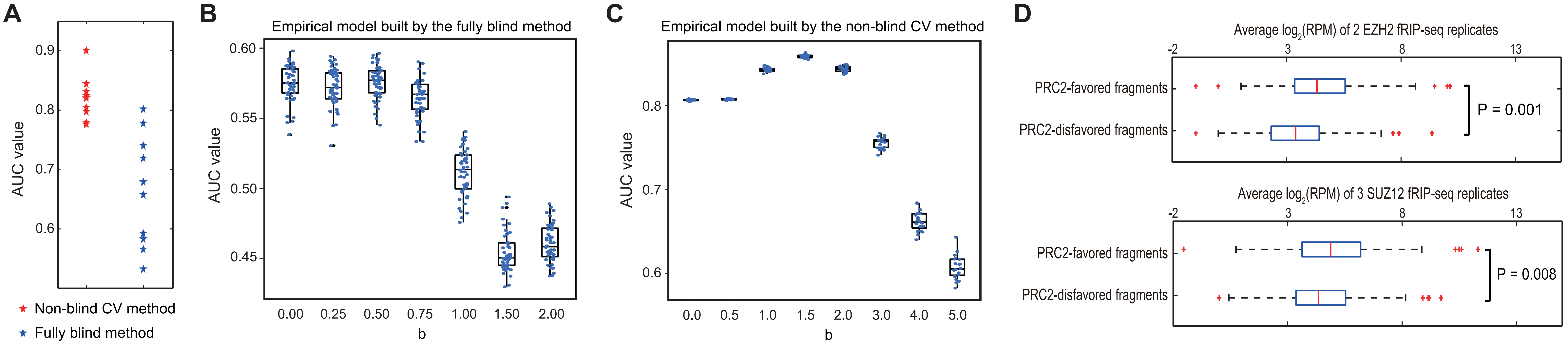

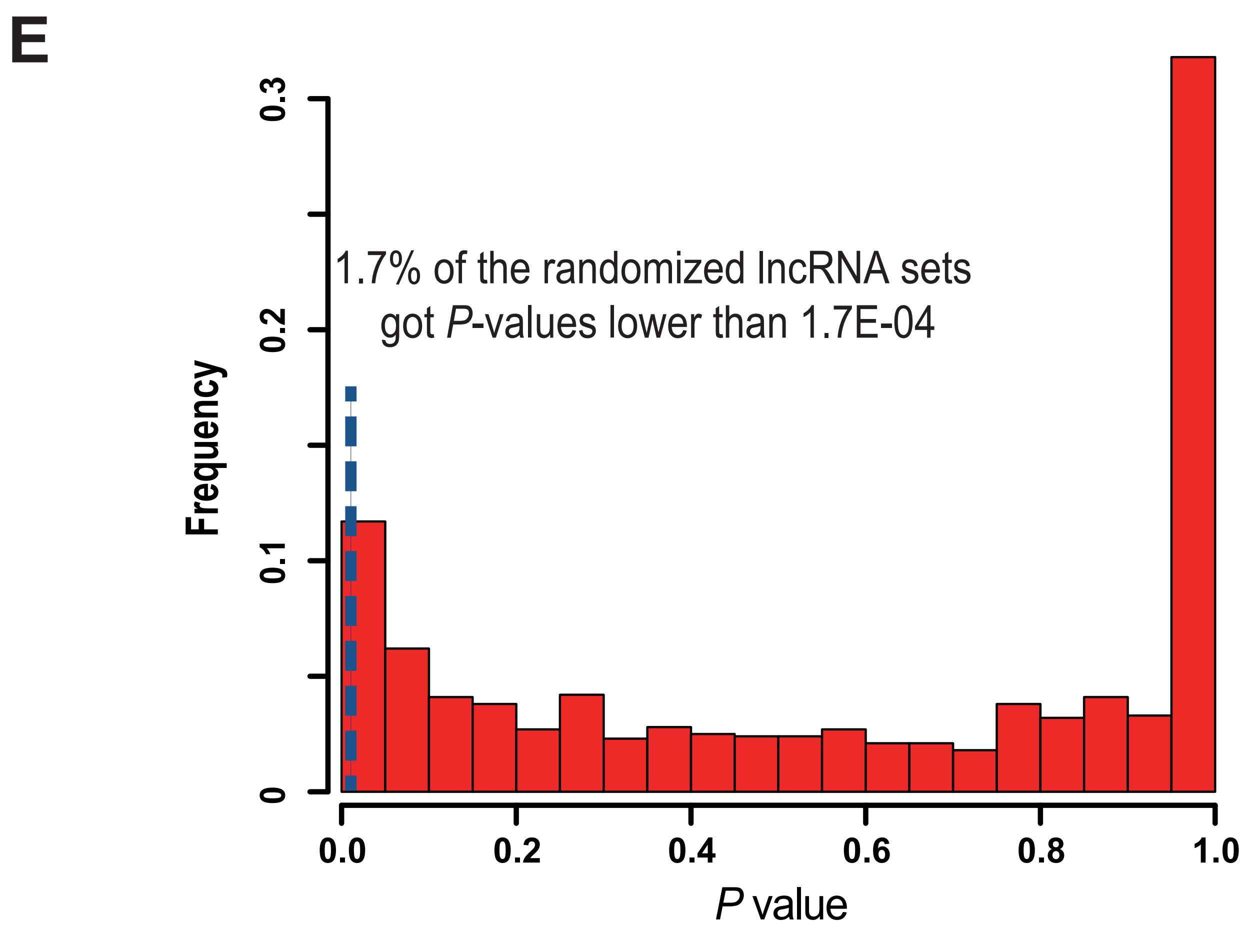

F

Test	Empirical *P*-value	FPR estimated from 1000 sets of randomized lncRNA
PRC2-favored fragments have higher average conservation score than PRC2-disfavored fragments (Figure 2C)	**1.7E-04**	**1.7%**
PRC2-favored fragments are more likely to contain conserved elements than the other parts of lncRNAs (Figure 2D)	**6E-04**	**0.8%**
PRC2-disfavored fragments are less likely to contain conserved elements than the other parts of lncRNAs (Figure 2D)	**2E-04**	**12.2%**

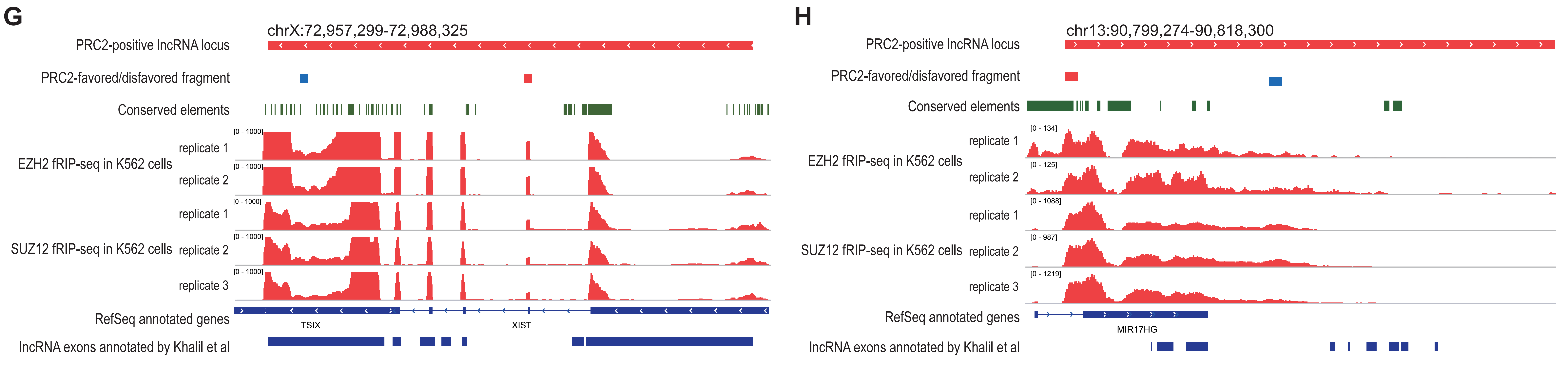

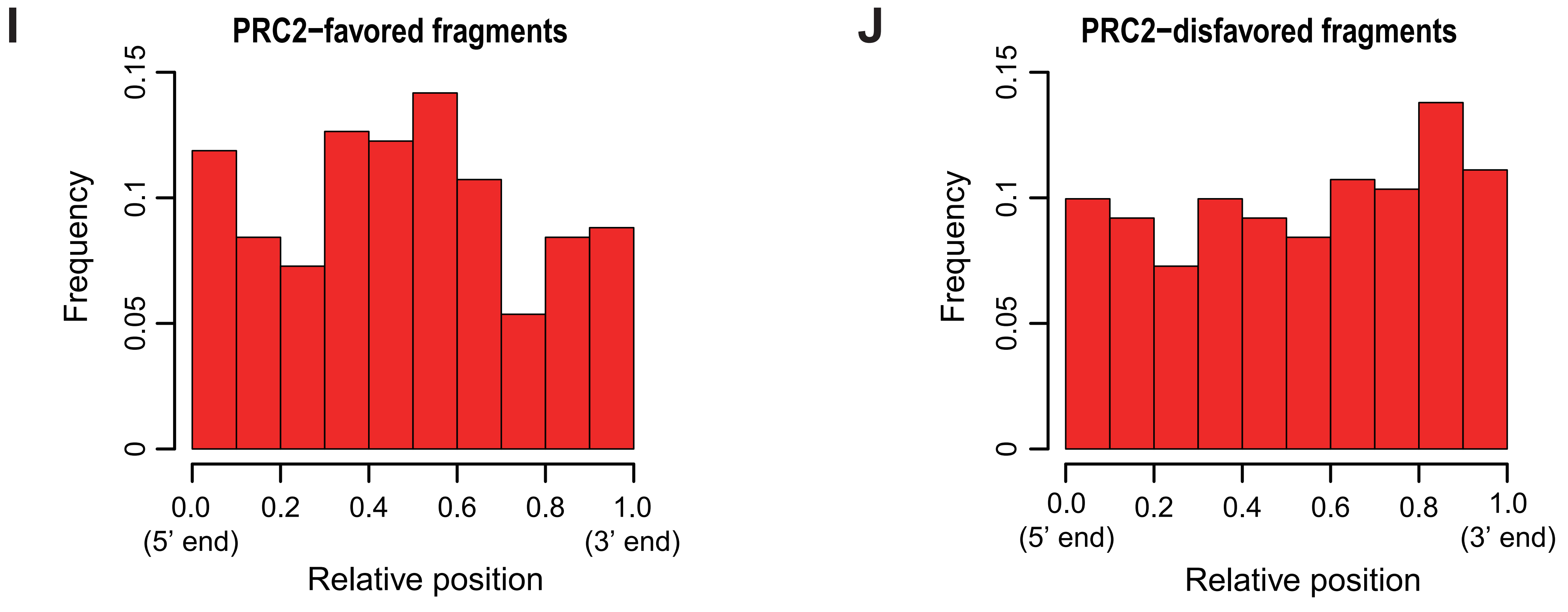

Supplementary Figure S2.

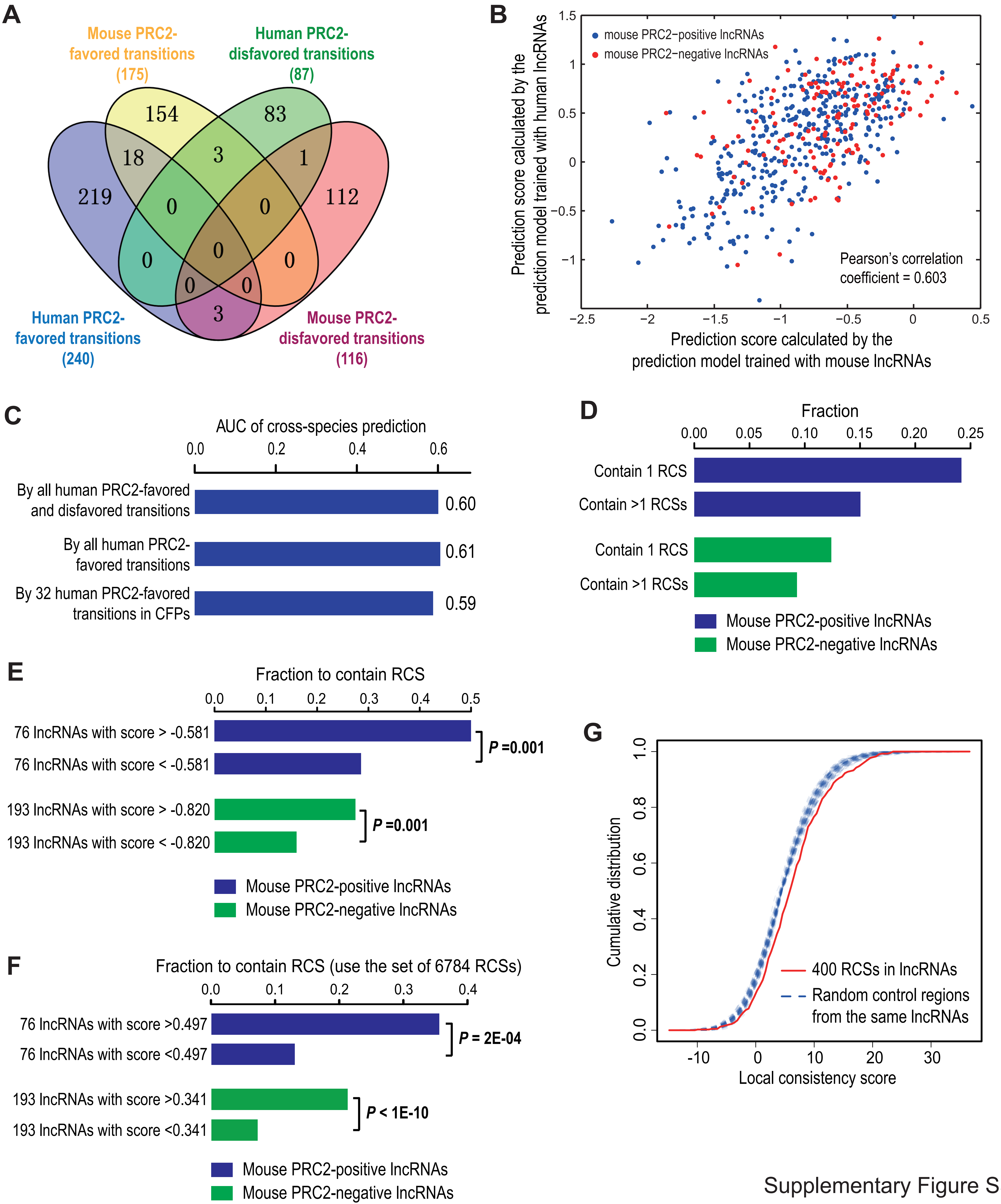

Supplementary Figure S3.

A

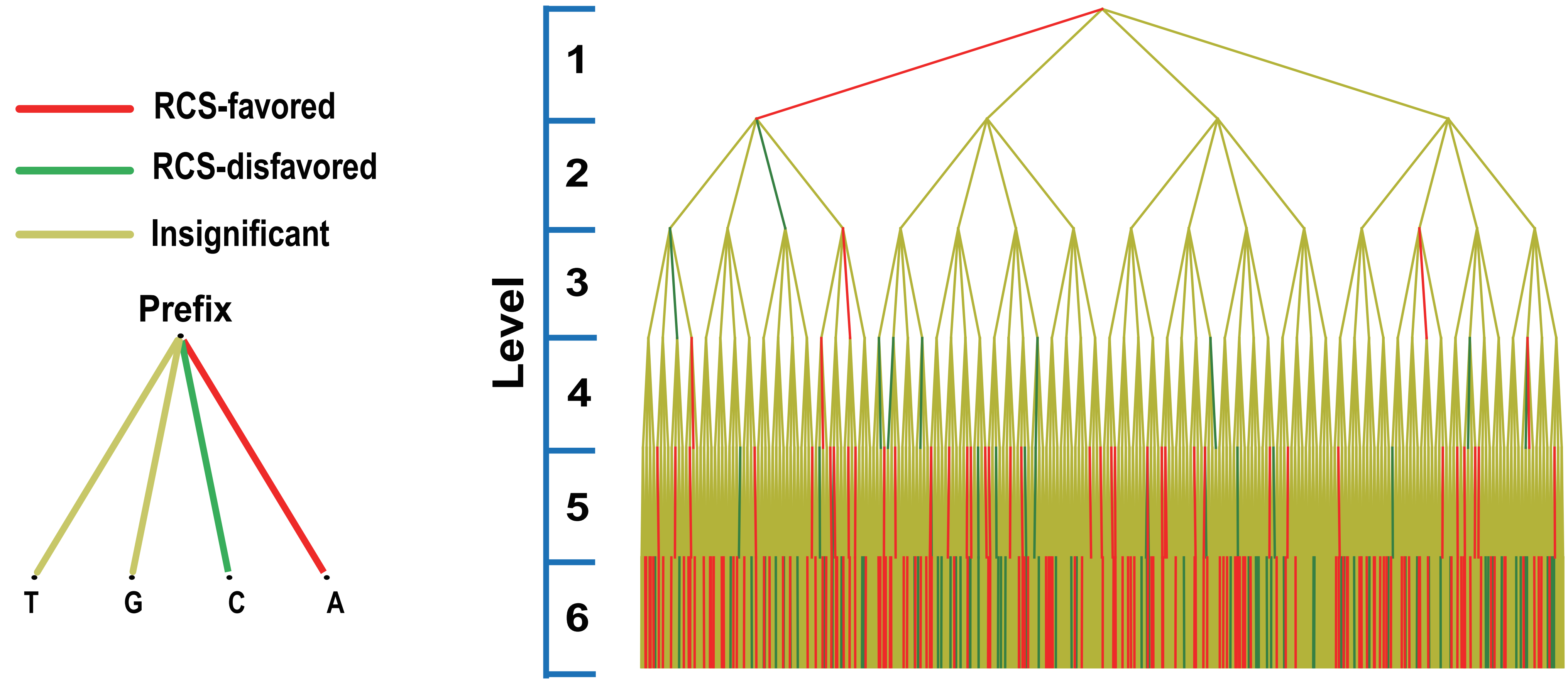

B

RCS-favored transitions	181
Fraction in CFPs	0.188
Average fraction in random permutations	0.062
P value	6E-06

C

RCS-disfavored transitions	99
Fraction in CDPs	0.101
Average fraction in random permutations	0.031
P value	0.013

D

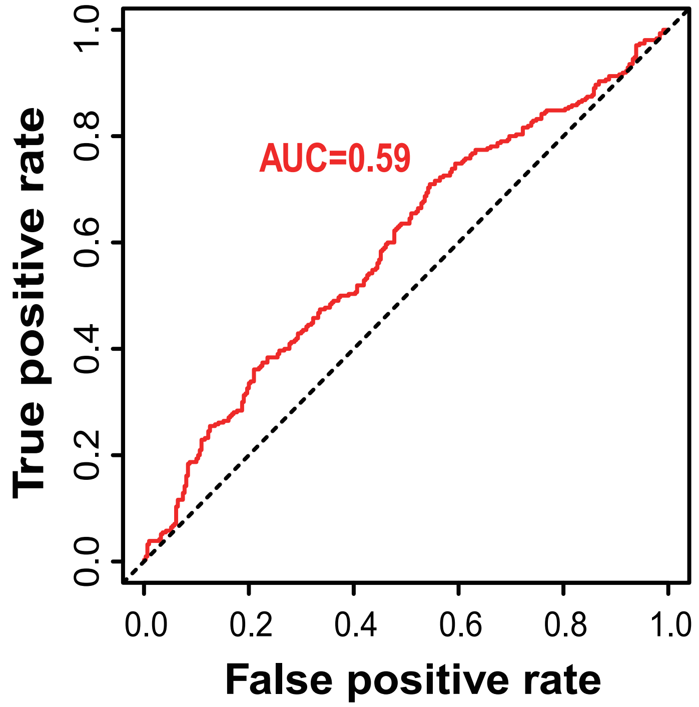

Supplementary Figure S4.

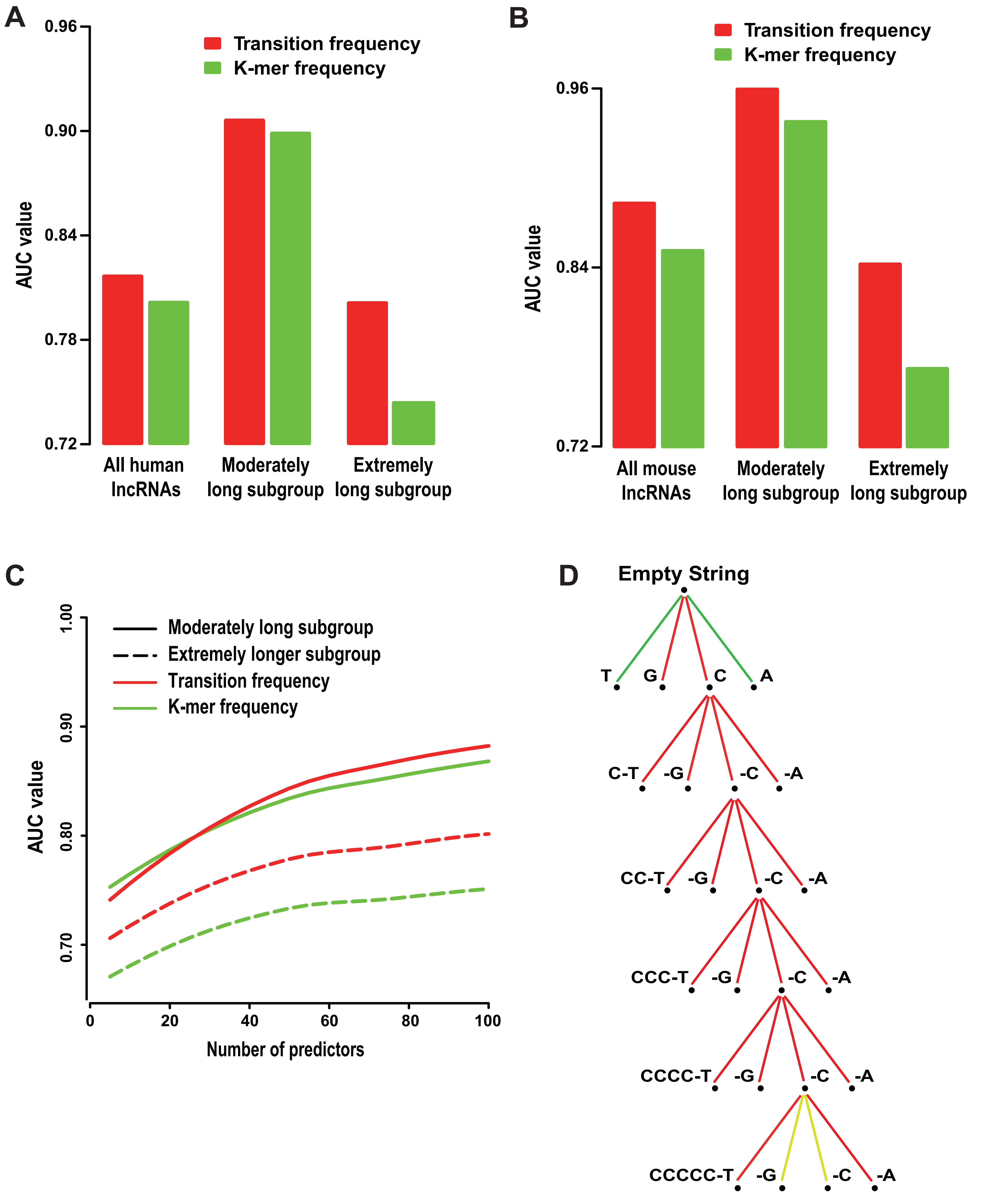

Supplementary Figure S5.

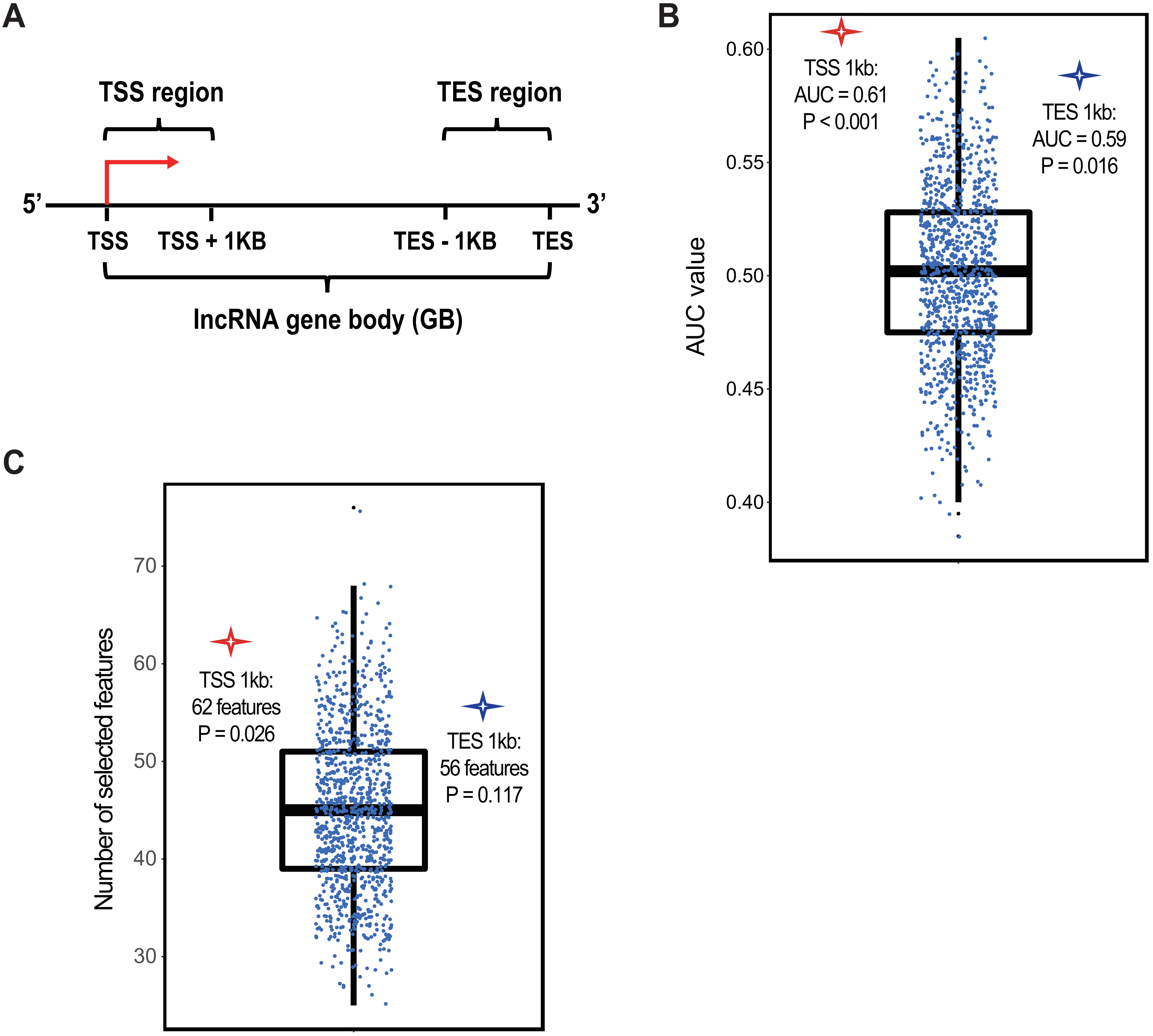

Supplementary Figure S6.
